# Bone biodeterioration—The effect of marine and terrestrial depositional environments on early diagenesis and bone bacterial community

**DOI:** 10.1371/journal.pone.0240512

**Published:** 2020-10-15

**Authors:** Anne Marie Høier Eriksen, Tue Kjærgaard Nielsen, Henning Matthiesen, Christian Carøe, Lars Hestbjerg Hansen, David John Gregory, Gordon Turner-Walker, Matthew James Collins, M. Thomas P. Gilbert

**Affiliations:** 1 Environmental Archaeology & Materials Science, Conservation & Natural Sciences, National Museum of Denmark, København, Denmark; 2 The GLOBE Institute, University of Copenhagen, København, Denmark; 3 Department of Plant and Environmental Science, University of Copenhagen, København, Denmark; 4 Department of Cultural Heritage Conservation, National Yunlin University of Science & Technology, Douliu, Yunlin County, Taiwan; 5 McDonald Institute for Archaeological Research, Cambridge, United Kingdom; 6 Norwegian University of Science and Technology, University Museum, Trondheim, Norway; 7 Center for Evolutionary Hologenomics, The GLOBE Institute, University of Copenhagen, København, Denmark; Universita degli Studi di Milano-Bicocca, ITALY

## Abstract

Bacteria play an important role in the degradation of bone material. However, much remains to be learnt about the structure of their communities in degrading bone, and how the depositional environment influences their diversity throughout the exposure period. We genetically profiled the bacterial community in an experimental series of pig bone fragments (femur and humeri) deposited at different well-defined environments in Denmark. The bacterial community in the bone fragments and surrounding depositional environment were studied over one year, and correlated with the bioerosion damage patterns observed microscopically in the bones. We observed that the bacterial communities within the bones were heavily influenced by the local microbial community, and that the general bone microbial diversity increases with time after exposure. We found the presence of several known collagenase producing bacterial groups, and also observed increases in the relative abundance of several of these in bones with tunneling. We anticipate that future analyses using shotgun metagenomics on this and similar datasets will be able to provide insights into mechanisms of microbiome driven bone degradation.

## 1. Introduction

Archaeological bones can provide valuable information about past vertebrates, including their diet, evolutionary relationships, demography and even health history (e.g. [1–3]). Thus, improving our understanding of the post mortem diagenetic processes that bones undergo and the interaction with the depositional environment is crucial for scientists wishing to better exploit them. Microbial bioerosion is one of the most commonly observed forms of bone degradation [4–6]. In this process, the microorganisms attacking the bone may leave characteristic tunnels in the bone microstructure that can be used to visually characterise the type of decay [7]. Two types of tunneling are commonly identified and used for characterisation, namely Wedl and Non-Wedl tunneling. Wedl tunneling is believed to be driven by aquatic microorganisms such as cyanobacteria, or fungal attack; whereas non-Wedl tunneling is believed to be caused by bacteria [7,8]. In theory, microbiological tools should allow those interested in bone bioerosion to obtain more nuanced insights into the diagenesis process. Although prior attempts have proven difficult, due to challenges in cultivating and identifying the communities [9] a few studies successfully managed to demonstrate that a selection of collagenase-producing soil bacteria can grow on bone, including members of the Firmicutes (e.g. *Bacillus subtilis*, *Clostridium* sp.) and Proteobacteria (e.g. *Pseudomonas* sp., *Alcaligenes* sp.) [10,11]. Fortunately, the past two decades has seen the emergence of DNA sequencing techniques such as metabarcoding and shotgun sequencing, that render it possible to genetically profile entire microbial communities within a sample.

Within forensic sciences, several studies have used genetic tools to examine the microbial community associated with decaying cadavers, in order to estimate the post-mortem interval (e.g. [12,13]). However, few studies have focused on the microbial community in articulated bones within cadavers, and fewer still on disarticulated bones. Nevertheless, one study used 16S rRNA gene metabarcoding to characterise the bacterial community in partly skeletonised human ribs [14], and found Proteobacteria to be the most dominant phylum across all samples, followed by Firmicutes, Bacteroidetes, Actinobacteria, Acidobacteria and Chloroflexi. Proteobacteria is a diverse phylum that is associated with any natural environment [15–18]. Subsequently, Damann and Jans [19] also used metabarcoding to explore the bacterial community of bones degrading within whole cadavers over a period of four years. These authors found that, while Firmicutes was the dominant phylum in bones degrading for less than one year, when bones were allowed to degrade for longer (1–4 years), Proteobacteria dominated the bacterial community. Both studies therefore reported a shift in the bacterial community over time after exposure. Taxonomic characterisation of the microbial composition in bones that have been buried, or left exposed articulated or as fragments, has to the best of our knowledge, not been attempted.

Several metabarcoding studies have also characterised the microbial community in soft tissues of cadavers as well as the surrounding environment [20–23]. In many of these studies a correlation was shown between the microbial community around a decomposing cadaver, and that within the local sediment, indicating that the sediment microbes could play a role in the degradation of the cadaver [13,24]. For example, Metcalf, *Xu et al*. [13] examined the microbial composition in the sediment prior to deployment of mouse and human cadavers, and found that approximately 40% of the microbial decomposer community originated from the sediment, although the sediment type itself was not a driving factor in the community development.

In this study, we aimed to contribute to the understanding of microbial degradation of bones during the early stages of diagenesis, and to examine how a marine or coast-near depositional environment influences this community. Unlike humans, bones from animal species typically consumed by humans predominantly enter into the archaeological record defleshed, and thus processed in some way. We therefore used an experimental sample set of defleshed raw, boiled and baked pig bones that were exposed in four different environments associated with submerged prehistoric archaeological sites. More specifically, we aimed to profile the microbial community composition over a time series using 16S metabarcoding as well as look for the presence and possible increases of collagenase producing bacteria within the tunneled bones.

## 2. Materials and methods

### 2.1 Study environments

The effect of bioerosion on bone exposed in four different depositional environments was studied, with all sites located within the temperate climate zone, in Denmark, Northern Europe (S1 Fig). Two of the depositional environments studied were marine burials placed in different types of sediment (submerged gyttja and submerged sand) 10–30 cm below the sea bed. The other two depositional environments were coastal, of which one was a burial at 30 cm depth in sand a few meters from the sea but permanently above sea water level (terrestrial sand), and the second was exposure on the ground in a tidal zone where the samples were flooded twice a day at high tide (tidal zone). In one environment (the submerged sand) we installed samples during both spring (S) and autumn (A), in order to investigate seasonal variation in the bacterial community.

A thorough environmental analysis of the depositional environments is presented in [25]. A summary of the environmental characteristics of the sites are shown in Table 1. In addition to these, two sediment cores (down to 50 cm) were collected from the two submerged environments for visual description and to measure the total organic content, grain size of the sediment and the sulphate and chloride content of the pore water. The sediment pore water was extracted using a Rhizon SMS (Rhizosphere, NL) attached to vacuum tubes via a syringe set, filtered at 0.45 μm, and the sulphate and chloride content was measured on an ion chromatograph (Thermo Fisher). Sediment samples were dried (at 105°C) and the total organic content was measured by loss on ignition (after ignition at 500°C) of the dried samples, where the loss was interpreted as the organic content. The grain size was measured on dried sediment samples (without removal of the organic content) on a Sieve Shaker (Buch & Holm A/S, Denmark) with eleven sieves ranging in mesh size 0.063–2 mm. The shaker was run at maximum speed for 2 min after the sample had been loaded onto the 2 mm sieve. The sediment caught in each sieve was weighted and the accumulated amount of sediment was calculated [26] and visually presented in a logarithmic scale graph.

**Table 1:** Summary of the environmental characteristics of the four depositional environments. Temperature and pH are reported as the median and range during the exposure period except when only a single measurement was available (indicated as n.m.).

Environment	Terrestrial sand	Tidal zone	Submerged gyttja	Submerged sand
*Temperature°C*	12.9 (3.3–19.6)	10.1 (0.6–20.9)	10.0 (0.1–18.3)	10.8 (0.7–20.5)
*pH*	7.3 (6.1–8.4)	8.8 (n.m.)	7.2 (6.8–7.6)	7.2 (7.0–7.7)
*Oxygen*	Oxic	Oxic	Anoxic	Anoxic
*Light*	No	Yes	No	No
*Water content*	Fluctuating	Fluctuating	Waterlogged	Waterlogged

### 2.2 Experimental setup

Fifty-six bone fragments were used in this study, all originating from either *femora* or *humeri* (three whole bones of each type) of domestic swine (*Sus scrofa domesticus*) obtained from a local butcher. We elected to use pig, for the practical reason that it is a larger mammal from which large quantities of similar bone types can be readily obtained and used without difficulty. As mentioned in the introduction, bones from animal species typically consumed by humans predominantly enter into the archaeological record defleshed, and processed in some way. To mimic this, the bones were either boiled (for 2 h in tap water), baked (in the oven at 200°C for 3 hours) or left simply defleshed (henceforth termed ‘raw’), prior to being cut into fragments of 2 x 2 cm. Details on sample preparation and installation are presented in Eriksen, Matthiesen *et al*. [25]. In short, two different systems were used for deployment in the depositional environments. At the marine sites the samples were secured on carbon fiber spears, and at two other sites the samples were sewed into fishing net and either buried or fastened on the ground. When designing the experiment, we deliberately chose to use bone fragments pretreated in this way, as it was consistent with similar material typically found in the Danish archaeological record. However, we acknowledge that in doing so, our samples were disconnected from the gut microbiome that could potentially influence the microbial community [27]. As such we caution against viewing our results as representative of buried material that is not defleshed (e.g. typical local human inhumations).

Following installation at the four depositional environments, samples were collected at 4, 14, 28 and 52 weeks (see S2 Fig for details on the sampling periods). At one site (the submerged sand), the study was twice (Autumn and Spring) disrupted after 28 weeks due to unforeseen construction work, thus the time series from this site is not for the full 52 weeks. Fragments were temporarily stored after collection at ca. 5–10°C in cool boxes (1-4h), until they could be stored frozen (-18°C). An overview of the samples is presented in Table 2. A few grams of sediment were collected at all the depositional environments in close proximity to the bone samples, at both time of installation, and time of retrieval of the bone samples. We additionally collected sediment for the tidal zone and submerged sand samples at a third period. Unfortunately, the sediment samples from the submerged gyttja environment subsequently defrosted due to a breakdown of the freezer they were stored in, thus we elected not to process them, as we deemed it likely that this would have distorted the true community profile in an unpredictable manner.

**Table 2:** Overview of samples used in the experiment. When no data is available, “nd” is reported. For the Scanning Electron Microscopy (SEM) imaging “None” is reported when the SEM analysis yielded no detectable tunneling, when Wedl attack is observed, “Wed” is reported in the table, while “bac” relates to non-Wedl microbial tunneling.

ID	Pretreatment	Environment	Exposure time (weeks)	SEM	#Reads before filtering	#Reads after filtering	#unfiltered ZOTUs
*20*	raw	Control	0	None	10709	7437	83
*9*	raw	Terrestrial sand	4	nd	2425	2298	95
*10*	raw	Terrestrial sand	14	nd	2120	2079	227
*11*	raw	Terrestrial sand	28	nd	87947	87316	1190
*12*	raw	Terrestrial sand	52	None	49614	49405	1420
*6*	raw	Tidal zone	14	nd	208092	207852	2614
*7*	raw	Tidal zone	28	nd	50839	50707	1205
*8*	raw	Tidal zone	52	Wed	195181	193579	3982
*1*	raw	Submerged gyttja	4	nd	241228	217896	476
*2*	raw	Submerged gyttja	14	nd	156777	152735	972
*3*	raw	Submerged gyttja	28	nd	64313	64127	562
*4*	raw	Submerged gyttja	52	None	129353	127610	1549
*13*	raw	Submerged sand (spring)	4	nd	84195	83856	398
*14*	raw	Submerged sand (spring)	14	nd	1126173	1052704	1477
*15*	raw	Submerged sand (spring)	28	nd	56953	52337	1443
*17*	raw	Submerged sand (autumn)	4	nd	111144	107584	894
*18*	raw	Submerged sand (autumn)	14	nd	120732	115678	751
*19*	raw	Submerged sand (autumn)	28	nd	93207	86430	503
*21*	Boiled	Control	0	None	14759	1336	45
*30*	Boiled	Terrestrial sand	4	nd	137500	137262	277
*31*	Boiled	Terrestrial sand	14	nd	117750	117052	1233
*32*	Boiled	Terrestrial sand	28	nd	38183	38131	1049
*33*	Boiled	Terrestrial sand	52	bac	819814	817121	3736
*26*	Boiled	Tidal zone	4	nd	33757	33725	1275
*27*	Boiled	Tidal zone	14	nd	32493	32462	1977
*28*	Boiled	Tidal zone	28	nd	49857	49833	1680
*29*	Boiled	Tidal zone	52	Wed	100833	96043	3155
*22*	Boiled	Submerged gyttja	4	nd	138580	137285	469
*23*	Boiled	Submerged gyttja	14	nd	98111	86902	875
*24*	Boiled	Submerged gyttja	28	nd	45450	42632	814
*25*	Boiled	Submerged gyttja	52	None	36423	35101	1105
*34*	Boiled	Submerged sand (spring)	4	nd	237401	236387	717
*35*	Boiled	Submerged sand (spring)	14	nd	98517	97975	722
*36*	Boiled	Submerged sand (spring)	38	None	87294	86451	1435
*38*	Boiled	Submerged sand (autumn)	4	nd	196378	188020	522
*39*	Boiled	Submerged sand (autumn)	14	nd	180136	169312	883
*40*	Boiled	Submerged sand (autumn)	28	nd	121835	99959	475
*41*	Baked	Control	0	None	3658	3297	65
*50*	Baked	Terrestrial sand	4	nd	249321	235708	377
*51*	Baked	Terrestrial sand	14	nd	245545	245482	857
*52*	Baked	Terrestrial sand	28	nd	107735	106871	1994
*53*	Baked	Terrestrial sand	52	None	151935	151260	3337
*46*	Baked	Tidal zone	4	nd	57872	57756	1320
*47*	Baked	Tidal zone	14	nd	93216	93202	719
*48*	Baked	Tidal zone	28	nd	97871	97794	2279
*49*	Baked	Tidal zone	52	Wed	562700	561684	6910
*42*	Baked	Submerged gyttja	4	nd	82808	82462	436
*43*	Baked	Submerged gyttja	14	nd	57087	55962	1027
*44*	Baked	Submerged gyttja	28	nd	100756	90923	716
*45*	Baked	Submerged gyttja	52	Wed	42336	41668	790
*54*	Baked	Submerged sand (spring)	4	nd	271449	270124	454
*55*	Baked	Submerged sand (spring)	14	nd	110663	108808	1632
*56*	Baked	Submerged sand (spring)	38	None	170111	159595	1261
*58*	Baked	Submerged sand (autumn)	4	nd	149706	145103	487
*59*	Baked	Submerged sand (autumn)	14	nd	127646	125271	840
*60*	Baked	Submerged sand (autumn)	28	nd	62732	59420	850
*82*	Sediment	Terrestrial sand	0	nd	552332	540883	5774
*93*	Sediment	Terrestrial sand	52	nd	687780	674948	6019
*80*	Sediment	Tidal zone	0	nd	954600	945656	8800
*83*	Sediment	Tidal zone	4	nd	1155354	1150669	6232
*85*	Sediment	Tidal zone	52	nd	32169	32077	3022
*81*	Sediment	Submerged sand	0	nd	1035338	1026415	10565
*86*	Sediment	Submerged sand	14	nd	33911	33459	2995
*88*	Sediment	Submerged sand	38	nd	827015	811393	9742
*61*	nd	Extraction blank	nd	nd	341	341	30
*62*	nd	Extraction blank	nd	nd	19	19	4
*N5*	nd	Extraction blank	nd	nd	18	18	6
*N6*	nd	Extraction blank	nd	nd	6	6	1
*37_b*	nd	Extraction blank	nd	nd	14	14	3
*57_b*	nd	Extraction blank	nd	nd	20	20	6
*Pbl*	nd	PCR negative	nd	nd	0	0	0

### 2.3 Scanning electron microscopy (SEM)

To visualise the potential microstructural damage to the bone fragments caused by microbial and environmental factors, a subset of fourteen of the bone fragments were dried in absolute ethanol, vacuum embedded in epoxy resin and prepared for Scanning electron microscopy (SEM) imaging as described in Turner-Walker [28]. Bones with the longest exposure times, as well as unburied controls, were used for the SEM studies.

### 2.4 DNA sampling and extraction

Post recovery, the bone fragments were visually inspected and photo documented (S1–S3 Figs in Eriksen, Matthiesen *et al*. [25]). Three control bone fragments (raw, boiled, baked) that had been maintained frozen throughout the experiment following initial pretreatment were also sampled. DNA was extracted from approximately 1000 mg of bone powder sampled (using a rotating drill (Dremel Europe, Bosch Power Tools, NL)) from each bone fragment, as well as 2500 mg of each of the eight sediment control samples (Table 2). Extraction blanks containing only the reagents from the extraction kit were also incorporated in the experiment. DNA was extracted from the bone powder following Eriksen, Matthiesen *et al*. [25] Text S3, which in brief involves an EDTA incubation to decalcify the samples prior to extraction using the PowerSoil® DNA Isolation Kit (Qiagen, US) following the manufacturer's protocol, with minor modifications (400 μL sample from above was added to the PowerBead Tubes). A Tissuelyser (Qiagen, Hilden, Germany) was used for 10 min at 30 Hz instead of a MO BIO Vortex Adaptor). Post extraction, the concentration was measured by Qubit^TM^ (dsDNA high sensitivity DNA assay, Thermo Fisher Scientific) and TapeStation (2200 TapeStation, Agilent Technologies, HS Assay).

### 2.5 Bacterial 16S metabarcoding

Prior to bacterial 16S metabarcoding, a subset of the DNA extracts was checked for the presence of PCR inhibitors using a qPCR assay based on a generic mammalian barcoding primer set (16Smam1 and 16Smam2), with PCR conditions described in Taylor [29]. The assay, which looked at how cycle threshold (Ct) values changed across a dilution series of each extract, showed no evidence of any PCR inhibition in the extracts. Subsequently, metabarcoding was principally performed as described in Eriksen, Puetz *et al*. [30] on the fifty-six bone extracts and the eight sediment extracts, using the 515F and 806R primers that amplify the V4 region of the bacterial ribosomal (rRNA) gene [31,32]. PCR products were visualised on a 2% agarose gel and subsequently pooled at roughly equimolar ratios as determined by gel band strength. Library construction on pooled amplicons was done using the Tagsteady protocol following Carøe and Bohmann [33]. Libraries were sequenced at the Danish National High Throughput Sequencing Center in Copenhagen, Denmark on an Illumina Miseq instrument using 300PE chemistry, with each pool getting ca. 15% of an Illumina MiSeq V3 flowcell (Illumina, US).

### 2.6 Processing sequence data

Unless otherwise specified, default parameters were applied in data processing. Overlapping paired-end Illumina sequencing reads were merged with USEARCH (v.10.0.240) [34]. Merged reads were demultiplexed with Cutadapt (v1.18) [35] and primers and barcodes were trimmed. After trimming, merged reads had an average length of 252 bp. Quality filtering of trimmed reads was performed with USEARCH using the -fastq_filter command with the -fastq_maxee_rate option set to 0.01 to remove reads with more than 1 error per 100 bases. Trimmed and filtered amplicon reads were denoised in USEARCH with the unoise3 command, which corrects sequencing errors and removes chimeric sequences. An OTU table with denoised zero-radius OTUs was made by mapping reads to OTUs (hereafter referred to as amplicon sequence variant, ASV). Taxonomy of the ASVs was predicted using SINTAX [36] against the SILVA database (v123) [37]. The resulting ASV table was imported into R [38] for downstream analyses. The ASV table was curated for erroneous ASVs using the LULU algorithm [39] and by removing ASVs that could not be taxonomically classified at Phylum level. Samples with fewer than 1,336 reads were removed. The following packages were applied in R for analyses: LULU, phyloseq [40], vegan [41], DESeq2 [42], PerformanceAnalytics [43], pheatmap [44], and ggplot2 [45].

For alpha diversity estimation, the Chao1 index was calculated on exposed bone samples only, using the “estimate_richness” function from phyloseq. A Pearson correlation analysis was performed using the base R function “cor” with Chao1 indices against sampling points for exposed bones. A Permanova test, using the “adonis” function from vegan package, with a Bray-Curtis distance matrix with pretreatment (boiled, baked, raw), environment, and exposure time as explanatory variables was performed to test what metadata contributed significantly to the variation within the dataset. Ordination with non-metric multidimensional scaling (NMDS from phyloseq function “ordinate”) was performed on ASV relative abundance counts, while canonical correspondence analysis (CCA from phyloseq function “ordinate”) was performed on a Bray-Curtis distance matrix with Hellinger-transformed ASV counts with the depositional environment as constraining variable.

ASVs that significantly differed in relative abundance between depositional environments were identified using the DESeq function, which identifies log2-fold changes. Of the ASVs varying significantly between depositional environments (p < 0.001), the 50 most abundant ones were extracted for plotting in heatmaps, where both ASVs and samples were hierarchically clustered on the axes.

Lastly, we undertook a preliminary analysis of association between bacterial taxonomy and decomposition of the bone material. Specifically, we looked for changes in abundance of bacteria in the bones deposited at the terrestrial sand, tidal zone and submerged gyttja environments, as these are the three depositional environments in which SEM analysis detected bacterial damage on the bone fragments. Based on the visual analysis of the type of damage observed in the bones from the marine versus the terrestrial environment, we assumed different, environment-specific, types of bacteria may have been active in the biodegradation, and attempted to identify candidate taxa that may be relevant for future study.

## 3. Results

### 3.1 Environmental analysis

The results from the organic content, grain size and sulphate:chloride analysis of the two submerged environments are shown in S3 Fig along with a brief visual characterisation. The results show that the upper 18 cm at the submerged gyttja environment consists of sandy gyttja with a high organic content (6–9% total organic matter) whereas deeper in the seabed (18–50 cm) the amount of gyttja decreases and the organic content is only 0–2%. The grain size is fine (<250 μm) to medium (<500 μm). As for the submerged sand environment, the upper 40 cm consists of fine sand with a few plant remains and an organic content below 1%, whereas below 40 cm the organic content increases to up to 3%. The grain size is very fine (<125 μm) and increases to fine (<250 μm) in the deeper layers. The results of the sulphate and chloride analysis suggest that sulphate reduction takes place in both environments. The submerged sand environment shows a linear drop in sulphate content down to 40 cm suggesting sulphate reduction at this depth, with sulphate being supplied from the seawater by diffusion through the top sediment. The submerged gyttja has a reduced sulphate content in the top sediment suggesting sulphate reduction in this part of the sediment profile.

### 3.2 Scanning electron microscopy

Overall, we observed signs of bioerosion (Wedl tunneling) in all the bones from the tidal zone after one year of exposure (S4 Fig), and in the baked bone fragment from the submerged gyttja environment (S5 Fig). In one case, we also observed bioerosion (non-Wedl tunneling) in the boiled bone fragment from the terrestrial sand environment (S7 Fig). We did not detect any attack on the bones from the submerged sand environment (exposed for 28 weeks), in the raw and baked bones from the terrestrial sand or in the raw and boiled bones from the submerged gyttja environment after one year of exposure. We also did not detect bioerosion on the unburied control bones.

Besides bioerosion, we also observed signs of chemical demineralisation and enlarged and ragged osteocyte lacunae, as well as enlarged canaliculi in the baked bone (S5 Fig) from the submerged gyttja environment. However, in general the bones from the two submerged environments were very well preserved, exhibiting only minor demineralisation (e.g. S6 Fig). Some caution is warranted because, as described in the methods section unforeseen construction work on the submerged sand depositional environment prematurely halted the experiment thus bones were only exposed for 28 weeks, as opposed to the full 52 weeks at the other environments.

The bones exposed for 52 weeks in the terrestrial sand showed signs of microbial tunneling entering from the periosteal border into a depth of approximately 200 μm (S7 and S8 Figs). The bones were also covered with closely adhering roots (Eriksen, Matthiesen *et al*. [25], S2–S4 Figs), which were clearly visible in secondary electron images of the unembedded bones, but which could not be observed directly in the cross-sections. However, local demineralisation that likely corresponded to root action, could be seen in the microstructural (SEM) analyses.

### 3.3 Metabarcoding results

From the bacterial metabarcoding data, we obtained a total of 13,399,719 raw unfiltered reads from the fifty-six bone extractions, eight sediment extractions, extraction negative controls and PCR negative control. In total, the negative controls had 0–20 reads, except for one extraction negative control which had 341 reads. The average number of reads per amplicon sample was 209,371 ranging from 2,120 to 1,155,345 (see Table 2). The total number of denoised ASVs was 25,778 prior to LULU curation of erroneous ASVs, of which 7278 were present in the bone fragments. The extraction negative control with the highest number of reads had 30 ASVs assigned, whereas the rest of the negative controls had 0–11 ASVs assigned. Based on the separate clustering in the NMDS ordination (S9 Fig), the negative controls were observed to cluster away from the exposed bone. Thus we believe that the source of contaminant DNA was low level templates in the reagents, rather than inter-extraction contamination, and that these contaminants were masked in the actual samples by the more abundant bacterial DNA present in them [46]. Therefore, they are unlikely to affect our conclusions. In Table 2, an overview of the number of raw reads (unfiltered) and untrimmed ASVs for each sample is presented.

#### 3.3.1 The bacterial community at the different environments

A Permanova test showed that sample environment (p = 0.001, R^2^ = 0.395) contributes most to the total dataset variation, while exposure time (p = 0.001, R^2^ = 0.084) also contributes significantly, although to a lesser degree. Pretreatment (boiled and baked) (p = 0.611, R^2^ = 0.0200) did not contribute significantly to the variation.

**Fig 1:**
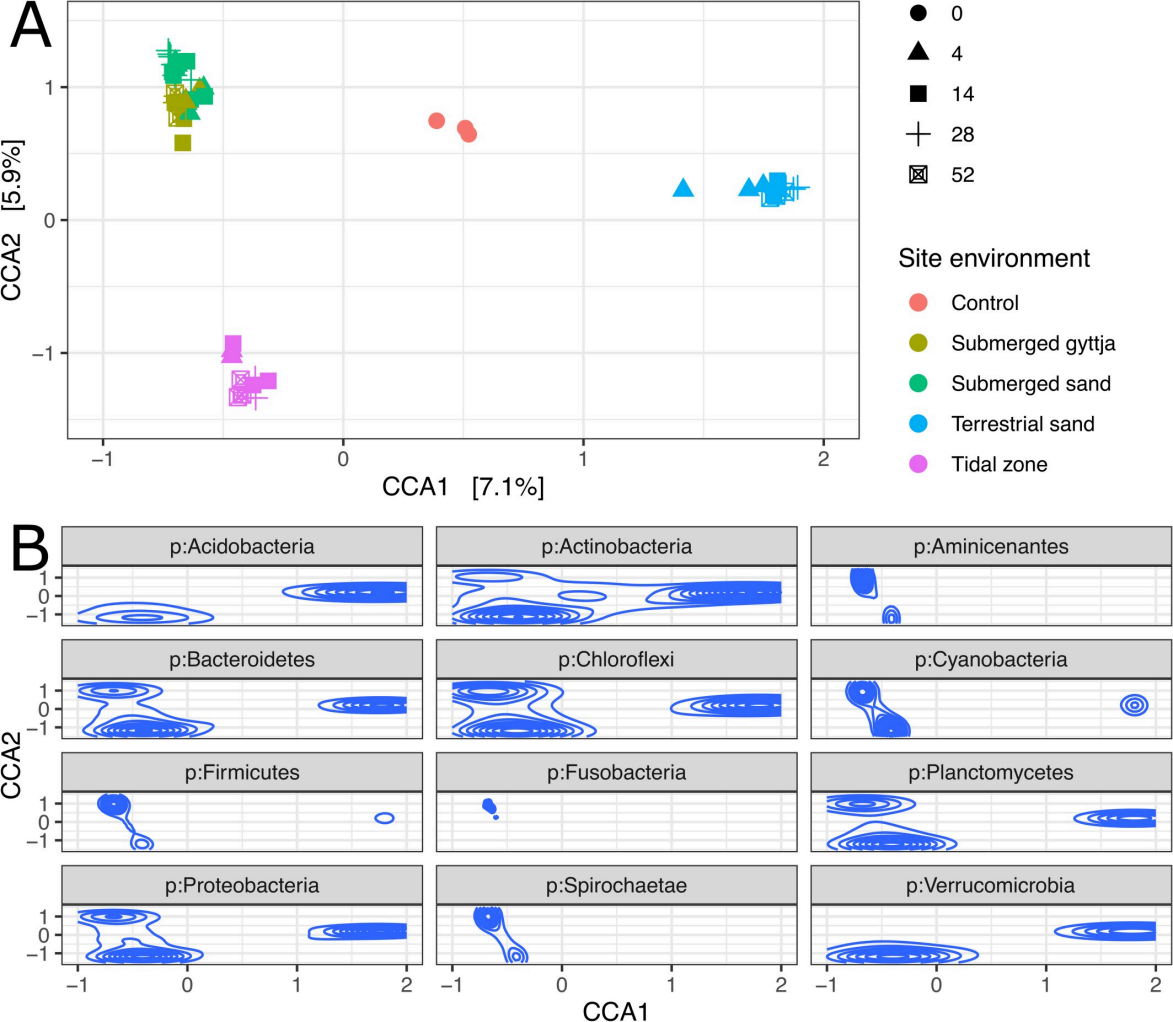
Canonical Correspondance Analysis (CCA) of the bone bacteria at the different environments. A) CCA analysis on Bray-Curtis distance matrix with Hellinger-transformed ASV abundances with the environment as a constraining variable. B) Below the main ordination are shown ASVs belonging to the top 12 most abundant phyla in 2D density estimates. Axes correspond to CCA1 and CCA2 in the main ordination, giving a representation of how the shown phyla influences the ordination of the samples. ‘Control’ refers to the unburied freezer-preserved bone fragments. 0, 4, 14, 28, 52 are the number of weeks the bones were deposited at the different environments.

A constrained correspondence analysis (CCA, Fig 1), with environment as a constraining variable shows how the bacterial community within the bones cluster by the depositional environment, with a distinct difference between the marine-associated (submerged sand, submerged gyttja and tidal zone), and the terrestrial environment, and the unburied control bones. The bacterial communities in the bones at the two submerged environments differ from the other environments, in having members of Fusobacteria and Firmicutes as major contributors to the bacterial community. The bone bacterial community at the two submerged environments and the tidal zone both contain members of the Aminicenantes and Spirochaetae. In general, however, regardless of environment, the bone bacterial communities of all samples are dominated by members of Actinobacteria, Bacteroidetes, Proteobacteria, Chloroflexi and Plantomycetes. This can also be seen in the relative abundance plot (S10 Fig). Cyanobacteria are also main contributors to the ordination of the bone bacterial community in all bones (except the three control/unburied bones), including the ones from the terrestrial sand, which is not surprising given they are generally ubiquitous in soil [47] and cyanobacteria are present in all sediment samples (S11 Fig).

**Fig 2:**
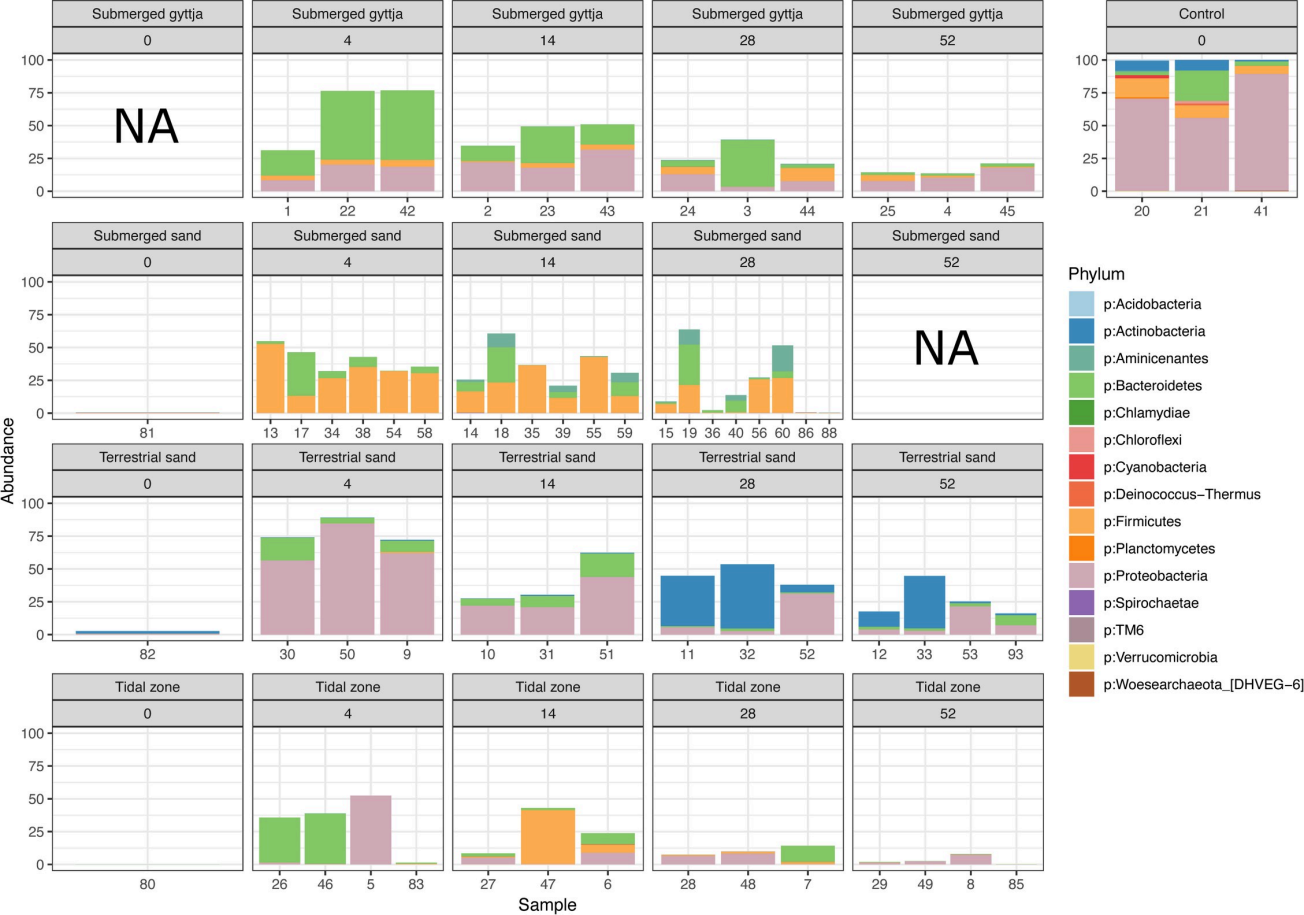
Bone bacteria at different time intervals. Relative abundance of ASVs, summarised at phylum level, present in the unburied control bone samples and their relative abundance in the sediment and exposed bone fragments (sample IDs on the x-axis are given in Table 2). Plots are subset by both their environment and the exposure time. The three unburied bones exhibit almost no variation and are dominated by ASVs belonging to Proteobacteria, followed by Firmicutes, Bacteroidetes and Actinobacteria. Pretreatment had no significant effect on the variation of the bacterial community (p = 0.611, R^2^ = 0.0200).

**Fig 3:**
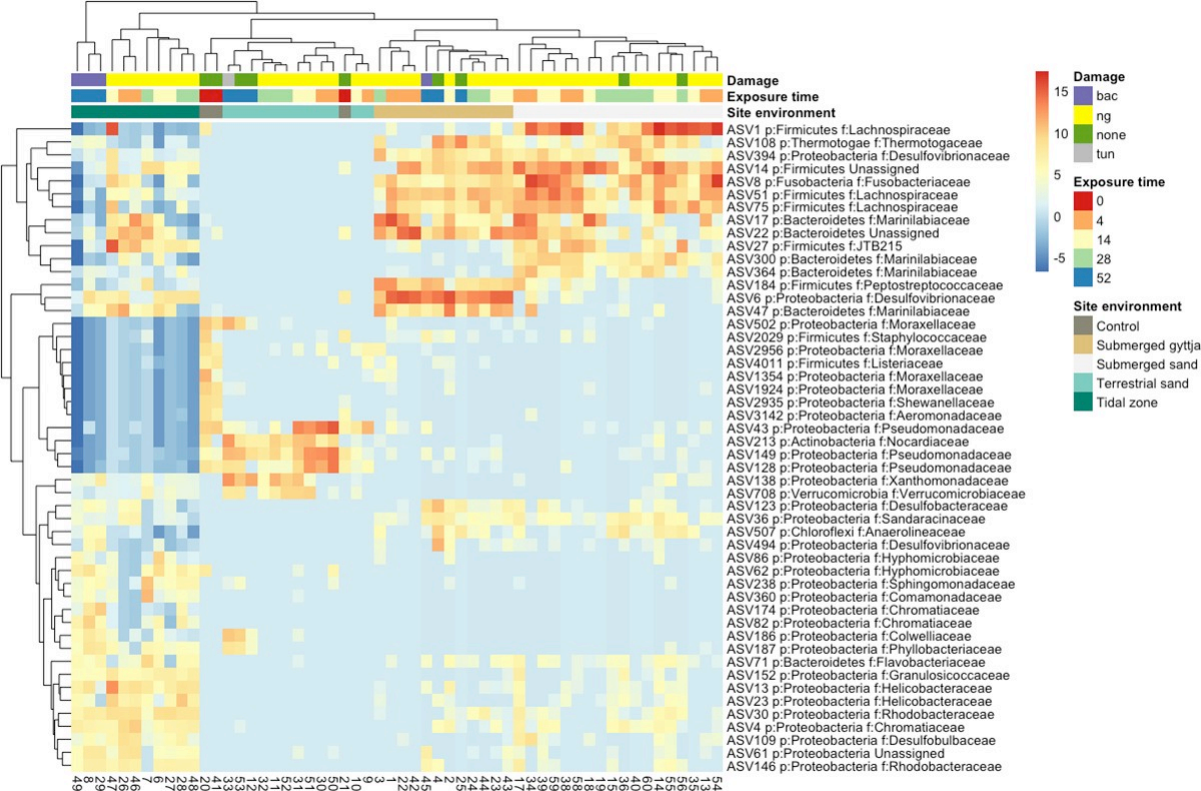
The most abundant bone bacteria at the different environments. Heatmap showing the log2 relative abundance difference of the top 50 most prevalent ASVs in the bone samples that display a significant difference in relative abundance between environments, as determined by the DESeq test. Taxonomy of ASVs are shown at phylum and family levels. Damage as observed in the SEM analysis and abbreviations are as follows: bac: non-Wedl-tunnels (in Table 2), nd: no data, none: tunnels absent, Wed = Wedl tunnels.

The bone bacterial diversity in the control samples (unburied bones) differs from that in the exposed samples, as the community in the latter could clearly be seen to be influenced by the bacterial community at the different depositional environments (Fig 1). The bacterial community in the bones from the terrestrial environment assembles the one in the control sample for the longest period (until week 28) before showing more similarities with the bacterial community found in the environment (Fig 2). The most abundant ASVs present in the unburied bones are only present at lower abundance and decreasing over time in the exposed samples (except for ASV2 belonging to Actinobacteria at the terrestrial sand environment which increases at 28 and 52 weeks) (Fig 3).

The differences in the bone bacterial community composition between depositional environments observed in the Permanova test and CCA plot (Fig 1), were further investigated in the DESeq test of differentially abundant ASVs between environments, and 1011 ASVs were found to be significantly different in their relative abundance between environments (p < 0.001). The top 50 most abundant ASVs that significantly differ in relative abundance between environments are shown in the heatmap (Fig 3). As observed above, the bacterial communities in bones from the submerged environments have many highly abundant ASVs in common. Here is it clear to see that the anaerobic Firmicutes family *Lachnospiraceae*, and especially the Bacteroidetes family *Marinilabiaceae*, contribute to the bacterial community in bone fragments at the submerged environments, alongside other anaerobic, sulphate-reducing or marine associated families of the phyla Fusobacteria (*Fusobacteriaceae*), Proteobacteria (*Desulfovibrionaceae*) and Thermotogae (*Thermotogaceae*). For the tidal zone samples, two Proteobacterial families (*Helicobacteraceae* and *Rhodobacteraceae)* contribute to the bone bacterial community, as well as several of the same families as seen in the two submerged environments (*Desulfovibrionaceae* and *Marinilabiaceae)*. Whereas for the terrestrial sand the Proteobacteria families *Pseudomonadacea* and *Xanthomonadaceae*, and the Actinobacteria family *Nocardiaceae* as well as the Verrrucomicrobia family *Verrucomicrobiaceae*, which contribute to the bone bacterial community.

#### 3.3.2 The bacterial community during the exposure period

**Fig 4:**
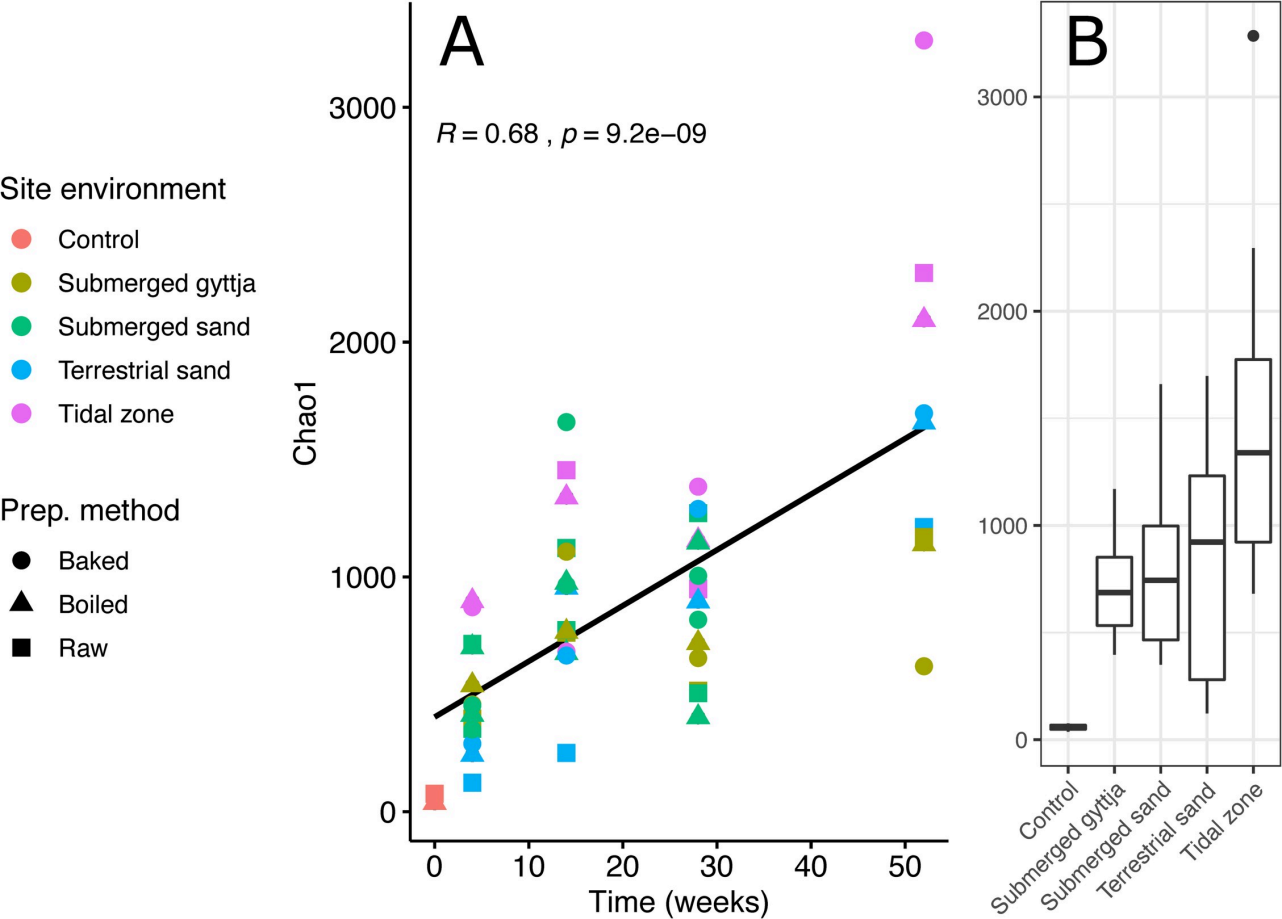
The diversity of bone bacteria over time. Alpha diversity (Chao1 index) plot as a function of time. A) A Pearson correlation coefficient shows that Chao1 correlates significantly and positively with exposure time. The regression line is shown with 95% confidence interval. B) Boxplot showing the diversity at each environment within all samples. Boxplots indicate interquartile range and median value as solid horizontal lines. Whiskers extend to 1.5 * of the interquartile ranges, and outliers are shown as circles.

Overall, the alpha diversity, as estimated with the Chao1 diversity index calculated on ASVs, of the bacterial community in the bone fragments was observed to generally increase over time (Fig 4; Pearson correlation test). Although this increase with time of the bacterial community diversity is observed in all bones from all environments, the alpha diversity was highest in the bones from the terrestrial sand and tidal zone environments.

#### 3.3.3 The bone degrading bacterial community

Our SEM imaging analyses revealed that all of the bones exposed for 52 weeks at the tidal zone (ID 8, 29, 49), and one bone from the submerged gyttja environment (the baked bone exposed for 52 weeks, ID 45) (Table 2, S4 and S5 Figs) showed similar damage, of a morphology that has previously been associated with Cyanobacteria [8,48]. We therefore analysed this cohort of samples to specifically look for both the presence of, and increases in the relative abundance of Cyanobacteria ASVs. Our analyses indicated that Cyanobacteria ASVs were indeed found in all the bones from the tidal zone, from the submerged gyttja, as well as the sediments in which each was buried. Furthermore, we observed that they had the highest relative abundance in the bones from the tidal zone (Fig 1). We also noted that while Cyanobacteria were absent in the unburied bones, they were present by time of first sampling (4 weeks) in all of the bones from the submerged gyttja and tidal zone.

We next decided to further explore the bone degrading bacterial community within the tunneled bones from the two marine associated environments, by conducting a Pearson correlation coefficient analysis on the relative abundance of ASV’s at these two environments. Phyla that significantly correlated with exposure time are shown in S1 Table.

We firstly noticed a significant increase in relative abundance occurred with time following exposure, in seventeen phyla from the tidal zone samples, and in twenty-seven phyla from the submerged gyttja environment. Eight of the phyla were shared between the two environments (Nitrospirae, Planctomycetes, Lentisphaerae, Candidate division OP3, Hyd24-12, Chloroflexi, Latescibacteria and Deferribacteres). Notably, none of these phyla are among the most relative abundant in the relevant environmental control samples (S11 Fig).

Secondly, we observed that the most abundant ASVs in the bone from the terrestrial sand in which we observed bacterial tunneling (the boiled bone exposed for 52 weeks, ID 33) were the Actinobacteria genera *Streptosporangium* and *Streptomyces* (S12 Fig). Several observations suggest that *Streptosporangium* may be of potential relevance for bone degradation in this environment. Firstly, although it is present at low frequency in both the unburied boiled control bone and the terrestrial sand environment itself (but absent in the other environments), its relative abundance increases in all the bones following exposure in the terrestrial sand environment. Secondly, its relative abundance is highest in the bone sample in which we observed bacterial tunneling.

Thirdly, based on previous studies on collagenase producing bacteria [49,50] we specifically investigated all the bones for the genera *Bacillus*, *Pseudomonas*, *Bacteroides* and *Clostridium*, along with the Alcaligenaceae family. The Firmicutes genus *Bacillus* was only present in the sediment at the terrestrial sand environment, as well as at very low abundance in the bones from that environment. However, no correlation between the bone that was tunneled, and the relative abundance of *Bacillus* was observed. The Proteobacteria *Pseudomonas* was observed in relatively high abundance in the unburied bones, and in the bones from the terrestrial sand environment. However, it decreased over time in the bones while increased in the sediment. It was only observed, in relatively low abundances, in the bones from the marine associated environments. The relative abundance of both the Bacteroidetes genus *Bacteroides* and the Firmicutes genus *Clostridium* within the bones decreased over time during exposure. They were not present in the unburied bones, but observed after 4 weeks of exposure in all bone fragments, but the relative abundance was highest in the bones from the three marine associated environments, only to decrease over time after exposure. The Proteobacteria family Alcaligenaceae were only present in the terrestrial bone and sediment, and were found at highest relative abundance in the one bone fragment that showed tunneling from this environment.

## 4. Discussion

### 4.1 Environmental analysis

Table 1 sums up the environmental conditions at the four depositional environments. While the temperature and pH values are similar, there are fundamental differences in the access of light, oxygen and sulphate at the four sites. All three parameters may influence the activity of the microbial fauna (and thus also the degradation of the bone fragments) by providing energy for their metabolism. The terrestrial sand environment has ample oxygen supply, but no light and only limited amounts of sulphate-rich seawater reaches these bone fragments. The fragments in the tidal zone are exposed to both light, oxygen and seawater. As for the two submerged environments they are not exposed to light or oxygen, but have an ample supply of sulphate and the results indicate a substantial sulphate reduction is ongoing in the most organic sediment layers (S3 Fig). Regarding the difference between the two submerged environments the gyttja environment is a well-known archaeological site [51,52], where the archaeology is preserved in the organic gyttja layers. The site is known to be under erosion [53] and the gyttja layers are relatively thin. The bone fragments were buried where only the upper 18 cm were very organic, below which the gyttja content decreased (S3 Fig). The bones were buried at 5–30 cm depth and thus they may have experienced different organic contents. This intra-site variation is not observed at the submerged sand environment where the upper 40 cm of the sediment are more homogenous. At both submerged environments the uppermost bone fragments will be most prone to degradation, both due to a faster supply of sulphate from the seawater above, and due to the risk of erosion of the sediment.

### 4.2 SEM

We observed bacterial damage on bone fragments exposed for one year, at three out of four depositional environments. In the fourth (the submerged sand) environment, the longest time the bones were exposed was 28 weeks (due to harbour construction work, we lost the rest of the samples at this site), and in those we did not detect any microbial damage (S6 Fig). In the tidal zone fragments and in one fragment (baked) from the submerged gyttja environment, we observed microbial attack (S4 and S5 Figs). Previous studies on bone or tooth submerged in a marine environment have found similar tunneling patterns [54–56]. Thus, finding this kind of tunneling in the bones deposited in a marine setting is not in itself surprising. However, the speed in which we observe the damage is interesting. Previous studies have suggested that microbial tunneling does not appear until after four-five years of exposure in a marine environment in the temperate climate zone [57] although in tropical freshwaters tunneling by aquatic microorganisms can appear after only six months [28].

Due to the initial installation design of the experiment, the baked bone fragments at the two submerged environments were buried shallower than the boiled and raw fragments (See Eriksen, Matthiesen *et al*. [25] Fig 1C). The baked bone fragment from the submerged gyttja environment in which we observe tunneling could have been exposed, or partly exposed, in the water column, potentially making it available for attack by organisms favouring higher oxygen or light levels. This assumption is supported by the aforementioned observations that the whole area is under erosion [53] and thus exposure of the bones during the experimental period is not implausible. This highlights the potential damage cultural heritage artifacts may experience if exposed to the light, oxygenated seawater and the bacteria herein.

At the terrestrial sand environment, we observed irrefutable non-Wedl tunneling in one (boiled) bone fragment after only one year’s exposure (S7 Fig). Interestingly, most of the tunneling was confined to a region between 50–200 μm beneath the surface (S8A Fig). This is a common pattern in archaeological bones where the surfaces in contact with the surrounding sediment are relatively un-tunneled compared to the interiors [9]. Perhaps significantly, we observe tunneling that breaches the periosteal surface of the bone at several different locations in this sample (S8B–S8E Fig). The very similar sizes and morphologies of these features suggest a common cause. Although this could be interpreted as simply where advancing bacterial colonies have travelled outwards from the interior and penetrated the surface from below this seems unlikely, considering these tunneling bacteria are generally found to avoid bone tissues close to the bone-soil interface. We therefore suggest that these represent where bacteria have entered the bone from the surrounding sediment and further speculate that the bone degrading bacterial community is to be found among the sediment bacteria.

### 4.3 Bacterial community

Overall, we first explored our dataset by looking for differences in the bacterial community within the bones correlated with environment, exposure time and pretreatment in order to investigate the variation of the bacterial community. We then used the data from the visual analysis as well as previous studies on collagenase producing bacteria to get a better understanding of the bone degrading bacterial community.

#### 4.3.1 Environment

Our results show that the bone depositional environment contributed significantly to the variation of the bone bacterial community. This indicates that the main contributors to the bacterial community within the bones during degradation were driven by the environment and parameters such as presence or absence of light, oxygen and sulphate, and that the bacterial community within the bones gives an indication of which type of environment the bones have been deposited in. The main contributors to the bone bacterial community at the anoxic environments (submerged gyttja and submerged sand) were obligately anaerobic bacteria (Fusobacteria (*Fusobacteriaceae*), Firmicutes (*Lachnospiraceae*) and especially the Bacteroidetes family *Marinilabiaceae*) (Figs 1 and 3). The two submerged environments were chosen due to their differences in organic content, grain size as well as sulphate:chloride content, to symbolise the degradation within two different kinds of anoxic, sulphate reducing environments. However, the genetic analysis of the bacteria shows that the largest overlap in the bone bacterial communities are between the two marine anoxic environments (submerged sand and submerged gyttja) where light and oxygen are absent. We found that obligate anaerobic bacterial families are main contributors to the bacterial community in the bones at these two environments, and that they share members of Spirochaeta and Aminicenantes with the bones from the tidal zone (Figs 1 and 3).

The bacterial community at the tidal zone is more closely related to the submerged environments than with the terrestrial bacterial community. The marine associated environments all have members of Spirochaeta contributing to the bacterial community. This diverse group of bacteria is widespread in nature, where members occur in a variety of aquatic environments including mud, ponds, lakes, rivers and oceans [58]. Free-living Spirochaeta are facultative anaerobes, which as with Fusobacteria fits with the depositional environments in which the bones were exposed. Aminicenantes are also a very diverse group of bacteria. They have been found across a wide range of salinity and temperature gradients, and it is suggested that they are anaerobic, and capable of fermenting carbohydrates and proteinaceous substrates [59]. Observations from previous studies [14,49] exploring the microbial communities within degrading bones have found that the community predominantly consists of the phyla Proteobacteria, Actinobacteria, Firmicutes and Bacteroidetes, as well to a lesser degree Acidobacteria and Planctomycetes. These studies were done on bones from whole cadavers, however, we find some similarities in the community structure, as across all four environments we find that members of these phyla are the main contributors to the microbial community (Fig 1, S10 Fig). Although we see this similarity in the bacterial community in degrading bones from different environments on phylum level the overall variation of the bacterial community in the bones are environment specific. It seems that environmental parameters such as light, the presence of sulphate as well as oxygen are the driving factors on the composition of the bone bacterial community.

When a bone is exposed to heat, both chemical and physical properties are altered [60]. Prior to obtaining our results we speculated that the heating process would influence the bacterial community within the bones during degradation. This assumption was based on the fact that the degradation of collagen is known to be influenced by heat and water content [61,62] and we incorporated both heating with (boiling) and without (baking) water in our experiment. Heating of the bones is also known to increase the bone porosity, making it easier for the bacteria to access [48]. However, the Permanova test with pretreatment as a constraining variable showed that pretreatment did not contribute significantly to the variation of the bacterial community in the bones. From the SEM analysis, we see bioerosion on bones with both kinds of pretreatments (boiled, baked) as well as the raw bones from the tidal zone after one year of exposure. Our results indicate that the type of environment, rather than the pretreatment, influences presence/absence of bone bioerosion.

#### 4.3.2 Exposure time

Our results show that the time of exposure contributes significantly to the variation of the bacterial community (p = 0.001, R^2^ = 0.088), although less so than the depositional environment (p = 0.001, R^2^ = 0.296). Across all environments the diversity of the bacterial community increases over time during the first year of deposition in which we obtained data. After exposure for one year, the largest richness is observed at the tidal zone followed by the terrestrial sand (Fig 4). That the bacterial community in the bones increases during the exposure period is not surprising, as one would presume bacterial activity to increase during decomposition of the bone material.

We speculate that the presence of bacteria in the unburied bones could result from putrefaction happening shortly after death and prior to sampling. As a general tendency, throughout the dataset we observe that the most abundant ASVs present in the unburied bones are only present at very low abundance, if at all, in the exposed bones, and become less abundant during the exposure period (Fig 2). However, the eight most abundant ASVs shared between all three (raw, boiled, baked) unburied bones, are in four cases present in very high abundance in the terrestrial environments, and not at any of the other environments. These four ASVs are all attributed to *Pseudomonas*, a common Proteobacteria Gram-negative bacterial genus. Members of the *Pseudomonas* are known to exhibit both lipolytic enzymatic and collagenase abilities and therefore breakdown lipids and collagen [63,64].

#### 4.3.3 Bone degrading community

Identifying the bone-degraders within the bacterial communities detected in these bone samples is complex, not least when datasets are restricted to 16S metabarcoding (such as ours). We used two approaches to examine the bone degrading bacterial community. Based on the attack patterns observed from the SEM imaging, we firstly looked for known collagenase producing bacteria in the bones with tunneling. We secondly looked at the relative abundance and increases in specific bacterial phyla within the bones from the environments in which we observe damage. This was done based on Metcalf, Xu *et al*.*’s* (13) study of the microbial community in degrading cadavers of human and mice, where they searched specifically for decomposers defined as “…*microbes that differentially increased during decomposition*..”, and found that 40% of the microbial decomposers were detected in soil prior to the experiment.

Based on the SEM analysis we observed at least two kinds of bioerosion, Wedl and None-Wedl tunneling. The type of damage observed on bones from the marine associated environments (the tidal zone and submerged gyttja) in which we found bone tunneling (Table 2, S4–S7 Figs) is normally attributed to cyanobacterial attack [48]. With this in mind we screened the samples for the presence and possible increases in the relative abundance of Cyanobacteria. We found cyanobacteria to be highly abundant in the sediment samples from all environments as well as in the bone fragments after 4–14 weeks (not present in the unburied bones), however, the relative abundance of Cyanobacteria was highest in the tidal zone (both sediment and bones). It is also at the tidal zone we observe tunneling in all bones after one year (we did not visually examine the bones for attack earlier than one year, so the attack could have happened before this). The tidal zone environment is characterised by having both the presence of sulphate-rich seawater, oxygen and light, which is optimal conditions for Cyanobacteria [65]. The relative abundance of Cyanobacteria is also high in the one bone from the submerged gyttja environment in which we observed attack, however, not as much as in the tidal zone. Based on this, there seems to be a correlation between the relative abundance of Cyanobacteria and the presence of tunneling in the bones. However, as the relative abundance of Cyanobacteria are already high in the bones from the tidal zone at time of first sampling (after 4 weeks), it could be speculated that this kind of damage could be observed even earlier on the bones.

To further explore the possible bone degrading bacterial community at the two marine associated environments (the tidal zone and submerged gyttja) in which we found bone tunneling (Table 2, S4–S7 Figs) we conducted a Pearson correlation coefficient analysis on the relative abundance of ASV’s that significantly correlated with time. In doing so, we observed that eight of these phyla were shared between the two environments Nitrospirae, Planctomycetes, Lentisphaerae, Candidate_division_OP3, Hyd24-12, Chloroflexi, Latescibacteria and Deferribacteres. Members of all eight phyla have been associated with marine habitat or anoxic metabolic pathways and as such their presence in the bones seems highly associated with the depositional environment and not necessarily biodeterioration [66–68]. However, further investigation of these phyla using shotgun genomic data would enable us to explore the possible presence of collagenase activity or other relevant genes associated with bone degradation.

To explore the bone degrading bacteria in the boiled terrestrial sand bone (ID 33), we screened for the most abundant ASVs present after one year, as this is where we observe none-Wedl tunnelling. The genera *Streptomyces* and *Streptosporangium* (Actinobacteria) were the two most abundant genera in this bone. Both genera are extremely interesting as they are known to form hyphae-like structures that reassemble fungal hyphae while degrading organic compounds in the soil [69]. In one study, Zaremba-Niedzwiedzka and Andersson [50] found that Actinobacteria accounted for more than 75% of the bacterial reads from a Neanderthal bone, of which the majority were members of the *Streptomyces*. Interestingly, they did not observe any of the typical DNA damage patterns associated with ancient DNA in the *Streptomyces* DNA, thus they hypothesised that it must have been derived from living bacteria that were active in the bone degrading process. Both collagenase and protease activity have been identified in members of *Streptomyces* [50]. We also observed an increase in relative abundance for the Proteobacterial family Alcaligenaceae, although in general at a lower relative abundance than that of *Streptomyces* and *Streptosporangium*. However, previous research has shown that members of the Alcaligenaceae are capable of producing collagenase and can use bone as a substrate [10,49]. Although these observations may be interesting in terms of implying which bacteria acts in terms of the bone diagenesis process, given the dynamic nature of many bacterial species' genomes, and the limited taxonomic resolution of metabarcoding data, future studies using shotgun sequenced metagenome data would be needed to clarify the relevance of our observations.

Based on previous studies on collagenase producing bacteria we screened all of the bone fragments for the genera *Bacillus*, *Pseudomonas*, *Bacteroides* and *Clostridium*, along with the Alcaligenaceae family. It is interesting to notice from these results, that there is no relationship between the relative abundance of these genera/family, and tunneling of the bones from the marine associated environments. We acknowledge that screening our dataset for already known collagenase producing bacteria is not the whole story as we speculate that many more groups of bacteria still undescribed could potentially have collagenase active genes. However, looking for already known collagenase producing bacteria is a start which can be elaborated with shotgun data later. *Pseudomonas* was only observed in relatively low abundance in the bones from the marine associated environments, and although *Bacteroides* and *Clostridium* was observed in relatively high abundance within the bones, their presence decreased over time during exposure. We speculate that more than one wave of bone degrading bacteria is present during the early stages of bone diagenesis, with perhaps the first degrading the easily available collagen and lipid sources, and the second degrading the collagen embedded within the mineralised part of the bone structure. This could explain why tunnels are not visible until the second wave is present. Such a change in the microbial community has been explored in various studies attempting to use the microbial community to estimate a time since death interval. Over a longer time period (1–4 years), Damann and Jans [19] also found a change in the bone bacterial community over time. As mentioned earlier, these observations could be interesting in terms of bone degradation, however, while amplicon metabarcoding has its clear advantages being both cost effective, fast and used in a variety of studies making the genetic databases available more exhaustive, the approach still has its disadvantages [32]. With the advancement of full shotgun metagenomic sequencing techniques, the sequence bias experienced with amplicon sequencing and issues of underrepresentation of uncultivable microorganisms in the databases, and therefore difficulties in characterisation, is reduced [70], although shotgun sequencing has recently been found to underrepresent low or high GC phyla with up to two logs [71]. A natural next step with this study would be to look into the functions of the different bacterial strains and examine how the bacterial communities are associated with bone tunneling using a shotgun metagenomics approach.

An understanding of the early bioerosion pathways and the influences from the exposure environment on the composition of the microbial community is of great value in the interpretations of archaeological bone specimens, and in the understanding of the early diagenetic stages of bone bioerosion.

## 5. Conclusion

We investigated the bacterial community in bones exposed at four different well documented environments using 16S metabarcoding, and in parallel conducted a visual assessment of the microbial damage pattern using SEM microscopy. We found that the deposition environment contributed significantly to the bacterial community composition within the bones. Not surprisingly, we also observed an increase in the bacterial diversity within the bones over time after exposure, in all four environments, with the community in bones from the tidal zone and terrestrial environments showing the most inter-environmental diversity. Furthermore, we did not observe an effect of pretreatment (raw, boiled, baked) on the bone bacterial community. We observed microbial tunneling in a bone fragment from the terrestrial environment and aquatic microbial attack on all fragments from the tidal zone after one year of exposure which corresponds well with the genetic analysis. Our findings give an insight into the initial bacterial bone diagenetic pathways as well as contribute to the knowledge of the depositional environments' influence on the bacterial community within degrading bones.

## Supporting information

S1 Fig Map of depositional environments. The location of the four different depositional environments are shown on the map of Denmark. The submerged gyttja and tidal zone environments are located on the Island of Hjarnø in the Bay of Horsens in Eatern Jutland, whereas the submerged sand and the terrestrial sand environments are located at the entrance to Isefjord in Northern Zealand. Figure modified from S1 Fig, Eriksen, Matthiesen *et al*. [25].
(TIF)

S2 Fig Sampling period. The sampling period for the four environments. Each bar symbolises one month.
(TIF)

S3 Fig Environmental analysis results. Results from the grain size, organic content and Sulphate:Chloride analysis of the sediments at the two submerged environments. Total organic matter is presented as % of sediment dry weight. The Sulphate:Chloride content is presented as mol/mol. For the submerged gyttja, the visual characterisation showed: 0–18 cm dark brown/grey sandy gyttja, 18–50 cm dark grey sand with some gyttja where the gyttja content decreases with depth. For the submerged sand, the visual characterisation showed: 0–40 cm light grey fine sand with few plant remains, 40–50 cm dark sand with significant amount of plant remains.
(TIF)

S4 Fig SEM image. A): Raw bone fragment exposed for one year at the tidal zone (sample ID 8). Extensive Wedl-tunneling is observed on the periosteal surface. B): Tunneling may be more extensive than the images suggest since there have been some obvious losses by exfoliation of tunneled surfaces. All samples deployed for one year at the tidal zone showed a similar type of damage. C): In addition to the severe tunneling into the periosteal surfaces the bones also exhibit some sporadic bioerosion in parts of the spongy bone (white arrow). Sand particles within the spongy bone are indicated by an asterix. D): Detail of Wedl-type tunneling. E): local demineralisation around Haversian canals (yellow arrow).
(TIF)

S5 Fig SEM image. A): Baked bone fragment exposed at the submerged gyttja depositional environment for one year (sample ID 45). B): Chemical demineralisation and enlarged porosity near the periosteal surface has caused surface losses. C): Wedl-tunneling in spongy bone. D): Detail of Wedl-type tunneling.
(TIF)

S6 Fig SEM image. Raw bone fragment deposited at the submerged sand (spring) environment for 28 weeks (Sample ID 16). A): Mosaic showing the whole section. B): Periosteal surface showing adhering fine sediment particles (yellow arrow). C): Endosteal surface showing only minor demineralisation and erosion with adhering sediment (blue arrow). There are no signs of bioerosion in the sample.
(TIF)

S7 Fig SEM image. Boiled bone fragment deposited for one year at the terrestrial sand environment (Sample ID 33). A): Non-Wedl, sub-micron microbial tunneling is observed near the periosteal surface and extends to a depth of 200 μm. B): Detail of area indicated by white box in A) showing tunneled regions and large ragged pores where bone has been lost. C): Hypermineralised border around the tunneled region (white arrow). D): The ragged, irregular voids are where both dissolved mineral and bacterially degraded collagen have been washed out of destructive foci. These are quite distinct from the well-delineated Wedl-tunnels seen in S4 and S5 Figs.
(TIF)

S8 Fig SEM image. The same boiled bone fragment deposited for one year at the terrestrial sand environment (Sample ID 33) as in S7 Fig. A): Periosteal surface showing numerous foci of non-Wedl, sub-micron microbial tunneling clustered 100–200 microns below the surface. B): Detail of this area showing the periosteal surface where it seems bacteria from the sediment may be entering the bone structure. C,D,E): Very similar morphologies observed at various places along the periosteal surface of the bone fragment. While it may be argued that these images simply show a tunneled zone breaching the surface, the remarkable similarities in size and morphology suggest otherwise. In addition, even in heavily tunneled bones the destructive foci tend to be limited to the interior of the compact bone. The outer 100 microns of the tissues are often quite well preserved.
(TIF)

S9 Fig Beta diversity of bacterial community in bones, sediment and DNA negatives. NMDS plot based on beta diversity of the bacterial communities in the bone fragments showing how the extraction and PCR negatives (controls) are clustering away from the rest of the samples.
(TIF)

S10 Fig Relative abundance of bacterial phyla in the bones. Relative abundance of the 12 most abundant bacterial phyla from the raw, boiled and baked bones respectively. Numbers on the x-axis denote the sample no. from Table 2.
(TIF)

S11 Fig Relative abundance of bacterial phyla in the sediment. Relative abundance of the most abundant bacterial phyla from the sediment samples. Numbers on the x-axis is the sample no. (Table 2). At the submerged sand samples were collected after 0 (81), 14 (86) and 28 (88) weeks. At the Terrestrial sand samples were collected at 0 (82) and 52 (93) weeks, and at the tidal zone samples were collected at 0 (80), 4 (83) and 52 (85) weeks.
(TIF)

S12 Fig Relative abundance of *Streptosporangium* in all bone and sediment samples. ´control 0’ refers to the unburied bones, where the results from all three unburied bones are shown, however *Streptosporangium* was absent from the raw and baked unburied bone, thus the results shown here only exhibit the relative amount in the unburied boiled bone fragment. Caution should be taken when assessing the sediment data, as data was not obtained from all environments at all time points (see Table 2).
(TIF)

S1 Table Bacterial phyla in the tidal zone and submerged gyttja. Phyla with a significant correlation with exposure time are given for the two marine associated environments in which we observe tunneling on the bones after one year of exposure.
(TIF)

## References

[1] Lee-Thorp J, Sealy JC, van der Merwe NJ. Stable carbon isotope ratio differences between bone collagen and bone apatite and their relationship to diet. Journal of Archaeological Science. 1989;16(6):585–99.

[2] Margaryan A, Hansen HB, Rasmussen S, Sikora M, Moiseyev V, Khoklov A, et al Ancient pathogen DNA in human teeth and petrous bones. Ecology and Evolution. 2018;8(6):3534–42. 10.1002/ece3.3924 29607044 PMC5869295

[3] Shapiro B, Drummond AJ, Rambaut A, Wilson MC, Matheus PE, Sher AV, et al Rise and fall of the Beringian Steppe Bison. Science. 2004;306:1561–5. 10.1126/science.1101074 15567864

[4] Hedges REM. Bone diagenesis: an overview of processes. Archaeometry. 2002;44(3):319–28.

[5] Jans MME, Nielsen-Marsh CM, Smith CI, Collins MJ, Kars H. Characterisation of microbial attack on archaeological bone. Journal of Archaeological Science. 2004;31:87–95.

[6] Turner-Walker G, Syversen U. Quantifying histological changes in archaeological bones using BSE-SEM image analysis. Archaeometry. 2002;44:461–8.

[7] Hackett CJ. Microscopical focal destruction (tunnels) in ex-humed human bones. Medicine Science and Law. 1981;21(4):243–65. 10.1177/002580248102100403 7321807

[8] Booth TJ. Using bone histology to identify stillborn infants in the archaeological record. In: Gowland R, Halcrow S, editors. The Mother-Infant Nexus in Anthropoloty, Bioarchaeology and Social Theory. Switzerland: Springer Nature; 2020 p. 193–209.

[9] Kendall C, Eriksen AMH, Kontopoulos I, Collins M, Turner-Walker G. Diagenesis of archaeological bone and tooth. Palaeogeography, Palaeoclimatology, Palaeoecology. 2018;491:21–37.

[10] Balzer A, Gleixner G, Grupe G, Schmidt HL, Schramm S, Turban-Just S. In vitro decomposition of bone collagen by soil bacteria: The implications for stable isotope analysis in archaeometry. Archaeometry. 1997;39:415–29.

[11] Grupe G, Dreses-Werringloer U, Parsche F. Initial stages of bone decomposition: causes and consequences. In: lambert JB, Grupe G, editors. Prehistoric human bone—Archaeology at the molecular level. Berlin: Springer Book Archive; 1993 p. 257–74.

[12] Cai J, Guo Y, Zha L, Guo J. The role of the microbiome in PMI estimation. In: Ralebitso-Senior TK, editor. Forensic Ecogenomics—The Application of Microbial Ecology Analyses in Forensic Contexts. London: Academic Press; 2018 p. 113–31.

[13] Metcalf JL, Xu ZZ, Weiss S, Lax S, van Treuren W, Hyde ER, et al Microbial community assembly and metabolic function during mammalian corpse decomposition. ScienceXpress. 2015;351(6269):158–62. 10.1126/science.aad2646 26657285

[14] Damann FE, Williams DE, Layton AC. Potential use of bacterial community succession in decaying human bone for estimating postmortem interval. Journal of Forensic Sciences. 2015;60(4):844–50. 10.1111/1556-4029.12744 25808627

[15] Roesch LFW, Fulthorpe RR, Riva A, Casella G, Hadwin AKM, Kent AD, et al Pyrosequencing enumerates and contrasts soil microbial diversity. The ISME Journal. 2007;1(4):283–90. 10.1038/ismej.2007.53 18043639 PMC2970868

[16] Eckburg PB, Bik EM, Bernstein CN, Purdom E, Dethlefsen L, Sargent M, et al Diversity of the human intestinal microbial flora. Science. 2005;308(5728):1635–8. 10.1126/science.1110591 15831718 PMC1395357

[17] Ley R, Bäckhed F, Turnbaugh PJ, Lozupone CA, Knight RD, Gordon JI. Obesity alters gut microbial ecology. PNAS. 2005;102(31):11070–5. 10.1073/pnas.0504978102 16033867 PMC1176910

[18] Li M, Wang B, Zhang M, Rantalainen M, Wang SH, Zhou H, et al Symbiotic gut microbes modulate human metabolic phenotypes. PNAS. 2007;105(6):2117–22. 10.1073/pnas.0712038105 PMC2538887 18252821

[19] Damann FE, Jans MME. Microbes, anthropology and bones. In: Carter DO, Tomberlin JK, Benbow ME, Metcalf JL, editors. Forensic Microbiology. Chp 12 UK: John Wiley & Sons Ltd.; 2017 p. 312–27.

[20] Hauther KA, Cobaugh KL, Jantz LM, Sparer TE, DeBruyn JM. Estimating time since death from postmortem human gut microbial communities. Journal of Forensic Sciences. 2015;60(5):1234–40. 10.1111/1556-4029.12828 26096156

[21] Hyde ER, Haarmann D, Petrosino JF, Lynne AM, Bucheli SR. Initial insights into bacterial succession during human decomposition. International Journal of Legal Medicin. 2015;129:661–71. 10.1007/s00414-014-1128-4 25431049

[22] Siles JA, Öhlinger B, Cajthaml T, Kistler E, Margesin R. Characterization of soil bacterial archaeal and fungal communities inhabiting archaeological human-impacted layers at Monte Lato settlement (Sicily, Italy). Scientific Reports. 2018;8(1903):1–14. 29382933 10.1038/s41598-018-20347-8 PMC5789874

[23] Singh B, Minick KJ, Strickland MS, Wickings KG, Crippen TL, Tarone AM, et al Temporal and spatial impact of human cadaver decomposition on soil bacterial and arthropod community structure and function. Frontiers in Microbiology. 2018;8(2616):1–12. 10.3389/fmicb.2017.02616 PMC5758501 29354106

[24] Carter DO, Metcalf JL, Bibat A, Knight R. Seasonal variation of postmortem microbial communities. Forensic Science, Medine and Pathology. 2015;11:202–7. 10.1007/s12024-015-9667-7 PMC9636889 25737335

[25] Eriksen AMH, Matthiesen H, Kontopoulos I, Gregory D, Collins M, Gilbert MTP. Rapid loss of endogenous DNA in pig bone buried in five different environments. Archaeometry. 2020.

[26] CEN-Standard. Tests for geometrical properties of aggregates—part 1: determination of particle size destribution—sieving method. DS/EN933-1. 2011;DS/EN 933–1.

[27] White L, Booth TJ. The origin of bacteria responsible for bioerosion to the internal bone microstucture: results from experimentally-deposited pig carcasses. Forensic Science International. 2014;239:92–102. 10.1016/j.forsciint.2014.03.024 24763128

[28] Turner-Walker G. Early bioerosion in skeletal tissues: persistence through deep time. Neues Jahrbuch für Geologie und Paläontologie. 2012;265(2):165–83.

[29] Taylor PG. Reproducibility of ancient DNA sequences from extinct Pleistocene Fauna. Molecular Biological Evolution. 1996;13(1):283–5. 10.1093/oxfordjournals.molbev.a025566 8583902

[30] Eriksen AMH, Puetz L, Rocha C, Nielsen TK, Hansen LH, Gilbert MTP. Releasing the microbes from old bones: The effect of different extraction protocols on microbial community profiling. STAR: Science & Technology of Archaeological Research. 2020.

[31] Gilbert JA, Jansson JK, Knight R. The earth microbiome project: successes and aspirations. BMC Biology. 2014;12(69):1–4. 25184604 10.1186/s12915-014-0069-1 PMC4141107

[32] Ziesemer KA, Mann AE, Sankaranarayanan K, Schroeder H, Oxga AT, Brandt BW, et al Intrinsic challenges in ancient microbiome reconstruction using 16S rRNA gene amplification. Nature Scientific Reports. 2015;5(16498):1–19. 10.1038/srep16498 PMC4643231 26563586

[33] Carøe C, Bohmann K. Tagsteady: a metabarcoding library preparation protocol to avoid false assignment of sequences to samples. BioRxiv. 2020. 10.1111/1755-0998.13227 32663358

[34] Edgar RC. Search and clustering orders of magnitude faster than BLAST Bioinformatics Applications note. 2010;26(19):2460–1. 10.1093/bioinformatics/btq461 20709691

[35] Martin M. Cutadapt removes adapter sequences from high-throughput sequencing reads. EMBnetjournal. 2011;17(1):10–2.

[36] Edgar RC. SINTAX: a simple non-Bayesian taxonomy classifier for 16S and ITS sequences. BioRxiv. 2016.

[37] Quast C, Pruesse E, Yilmaz P, Gerken J, Schweer T, Yarza P, et al The SILVA ribosomal RNA gene database project: improved data processing and web-based tools. Nucleic Acids Research. 2013;41:590–6. 10.1093/nar/gks1219 PMC3531112 23193283

[38] Team RDC. R: A language and environmnet for statistical computing. R Found Stat Comput 2016.

[39] Frøslev TG, Kjøller R, Bruun HH, Ejrnæs R, Brunbjerg AK, Pietroni C, et al Algorithm for post-clustering curation of DNA amplicon data yields reliable biodiversity estimates. Nature Communication. 2017;8(1188). 10.1038/s41467-017-01312-x PMC5662604 29084957

[40] McMurdie PJ, Holmes S. Phyloseq: An R package for reproducible interactive analysis and graphics of microbiome census data. PlosOne. 2013;8(4):e61217. 10.1371/journal.pone.0061217 PMC3632530 23630581

[41] Dixon P. VEGAN, a package of R functions for community ecology. Journal of Vegetation Science. 2003;14(6):927–30.

[42] Love MI, Huber W, Anders S. Moderated estimation of fold change and dispersion of RNA-seq data with DESeq2. Genome Biology. 2014;15(550):1–21. 10.1186/s13059-014-0550-8 PMC4302049 25516281

[43] Peterson BB, Carl P, Boudt K, Bennett R, Ulrich J, Zivot E, et al Package PerformanceAnalytics. R Topic documented. 2020.

[44] Kolde R. pheatmap: Pretty heatmaps. R Package version version 108. 2015.

[45] Wickham H. ggplot2: Elegant Graphics for Data Analysis. 2016, Springer-Verlag New York. ISBN 978-3-319-24277-4.

[46] Salter SJ, Cox MJ, Turek EM, Calus ST, Cookson WO, Moffatt MF, et al Reagent and laboratory contamination can critically impact sequence-based microbiome analyses. BMC Biology. 2014;12(87):1–12. 25387460 10.1186/s12915-014-0087-z PMC4228153

[47] Abinandan S, Subashchandrabose SR, Venkateswarlu K, Megharaj M. Soil microalgae and cyanobacteria: the biotechnological potential in the maintenance of soil fertility and health. Critical Review in Biotecnology. 2019;39(8):981–998. 10.1080/07388551.2019.1654972 31455102

[48] Turner-Walker G. Light at the end of the tunnels? The origins of microbial bioerosion in mineralised collagen. Palaeogeography, Palaeoclimatology, Palaeoecology. 2019;529:24–38.

[49] Emmons AL, Mundorff A, Keenan SW, Davoren JM, Andronowski J, Carter DO, et al Patterns of microbial colonization of human bone from surface-decomposed remains. BioRxiv. 2019. 10.1371/journal.pone.0218636 PMC7343130 32639969

[50] Zaremba-Niedzwiedzka K, Andersson SGE. No ancient DNA damage in actinobacteria from the Neanderthal bone. PlosOne. 2013;8(5): e62799. 10.1371/journal.pone.0062799 PMC3643900 23658776

[51] Skriver C, Astrup PM, Borup P. Hjarnø Sund—all year, all inclusive. A submerged Late Mesolithic coastal site with organic remains. Danish Journal of Archaeology. 2018;7(2):195–217.

[52] Ward I, Moe-Astrup P, Merigot K. At the water's edge: Micromorphological and quantitative mineral analysis of a submerged Mesolithic shell midden at Hjarnø Sund, Denmark. Journal of Archaeological Science. 2019;102:11–25.

[53] Skriver C, Borup P, Astrup PM. Hjarnø Sund: An eroding Mesolithic site and the tale of two paddles. In: Bailey GN, editor. Under the Sea: Archaeology and Palaeolandscapes: Springer International Publishing; 2017.

[54] Arnaud G, Arnaud S, Ascenzi A, Bonucci E, Graziani G. On the problem of the preservation of human bone in sea-water. Journal of Human Evolution. 1978;7:409–20.

[55] Bell LS, Boyde A, Jones SJ. Diagenetic alteration to teeth in situ illustrated by backscattered electron imaging. Scanning. 1991;13:173–83.

[56] Turner-Walker G, Jans MME. Reconstructing taphonomic histories using histological analysis. Palaeogeography, Palaeoclimatology, Palaeoecology. 2008;266:227–35.

[57] Yoshino M, Kimijima T, Miyasaka S, Sato H, Seta S. Microscopical study on estimation of time since death in skeletal remains. Forensic Science International. 1991;49(2):143–58. 10.1016/0379-0738(91)90074-s 1855715

[58] Canale-Parola E. Motility and chemotaxis of Spirochetes. Annual reviews of Microbiology. 1978;32:69–99. 10.1146/annurev.mi.32.100178.000441 360979

[59] Kadnikov VV, Mardanov AV, Beletsky AV, Karnachuk OV, Ravin NV. Genome of the candidate phylum *Aminicenantes* bacterium from a deep subsurface thermal aquifer revealed its fermentative saccharolytic lifestyle. Extremophiles. 2019;23:189–200. 10.1007/s00792-018-01073-5 30600356

[60] van Hoesel A, Reidsma FH, van Os BJH, Megens L, Braadbaart F. Combusted bone: Physical and chemical changes of bone during laboratory simulated heating under oxidising conditions and their relevance for the study of ancient fire use. Journal of Archaeological Science: Reports. 2019;28(102033):1–13.

[61] Chowdhury MP, Wogelius R, Manning PL, Metz L, Slimak L, Buckley M. Collagen deamidation in archaeological bone as an assessment for relative decay rates. Archaeometry. 2019;61(6):1382–98.

[62] Lambri ML, Bozzano PB, Giordano EDV, Bonifacich FG, Gargicevich D, Zelada GI, et al Denaturation processes of collagen from cow bones as a function of temperature. Revista Materia. 2018;23(2).

[63] Jaeger KE, Kovacic F. Determination of lipolytic enzyme activities. In: Filloux A, Ramos JL, editors. Pseudomonas Methods and Protocols. 1149 New York: Springer Protocols; 2014 p. 111–34. 10.1007/978-1-4939-0473-0_12 24818902

[64] Schoellmann G, Fisher E Jr. A collagenase from *Pseudomonas aeruginosa*. Biochimica et Biophysica Acta (BBA)—Enzymology and Biological Oxidation. 1966;122(3):557–9.

[65] Spring S, Sorokin DY, Verbarg S, Rohde M, Woyke T, Kyrpides NC. Sulfate-reducing bacteria that produce exopolymers thrive in calcifying zone of a hypersaline cyanobacterial mat. Frontiers in Microbiology. 2019;10(862):1–19. 31068923 10.3389/fmicb.2019.00862 PMC6491731

[66] Daims H, Lebedeva EV, Pjevac P, Han P, Herbold C, Albertsen M, et al Complete nitrification by Nitrospira bacteria. Nature. 2015;528:504–9. 10.1038/nature16461 26610024 PMC5152751

[67] Fuerst JA, Sagulenko E. Beyond the bacterium: planctomycetes challenge our concepts of microbial structure and function. Nature Reviews. 2011;9:403–13. 10.1038/nrmicro2578 21572457

[68] Hedlund BP, Cho JC, Derrien M, Costa KC. Phylum XXII. In: Krieg NR, Staley JT, Brown DR, Hedlund BP, Paster BJ, Ward NL, et al, editors. BERGEY’S MANUAL® Systematic Bacteriology—The Bacteroidetes, Spirochaetes, Tenericutes (Mollicutes), Acidobacteria, Fibrobacteres, Fusobacteria, Dictyoglomi, Gemmatimonadetes, Lentisphaerae, Verrucomicrobia, Chlamydiae, and Planctomycetes. 4. USA: Springer; 2010 p. 785–94.

[69] Chater KF. Recent advances in understanding Streptomyces. F10000 Faculty Review. 2016;2795:1–16. 10.12688/f1000research.9534.1 PMC5133688 27990276

[70] Warinner C, Herbig A, Mann AE, Yates JAF, Weiss CL, H.A. B, et al A robust framework for microbial archaeology. Annual Review of Genomics and Human Genetics. 2017;18:321–56. 10.1146/annurev-genom-091416-035526 28460196 PMC5581243

[71] Browne PD, Nielsen TK, Kot W, Aggerholm A, Gilbert MTP, Puetz L, et al GC bias affects genomic and metagenomic reconstructions, underrepresenting GC-poor organisms. GigaScience. 2020;9:1–14. 10.1093/gigascience/giaa008 PMC7016772 32052832

