# Supplementary figures and images for: Bone biodeterioration—The effect of marine and terrestrial depositional environments on early diagenesis and bone bacterial community
Source: PLoS One. 2020 Oct 15;15(10):e0240512. doi: 10.1371/journal.pone.0240512 (PMC7561151; doi:10.1371/journal.pone.0240512)

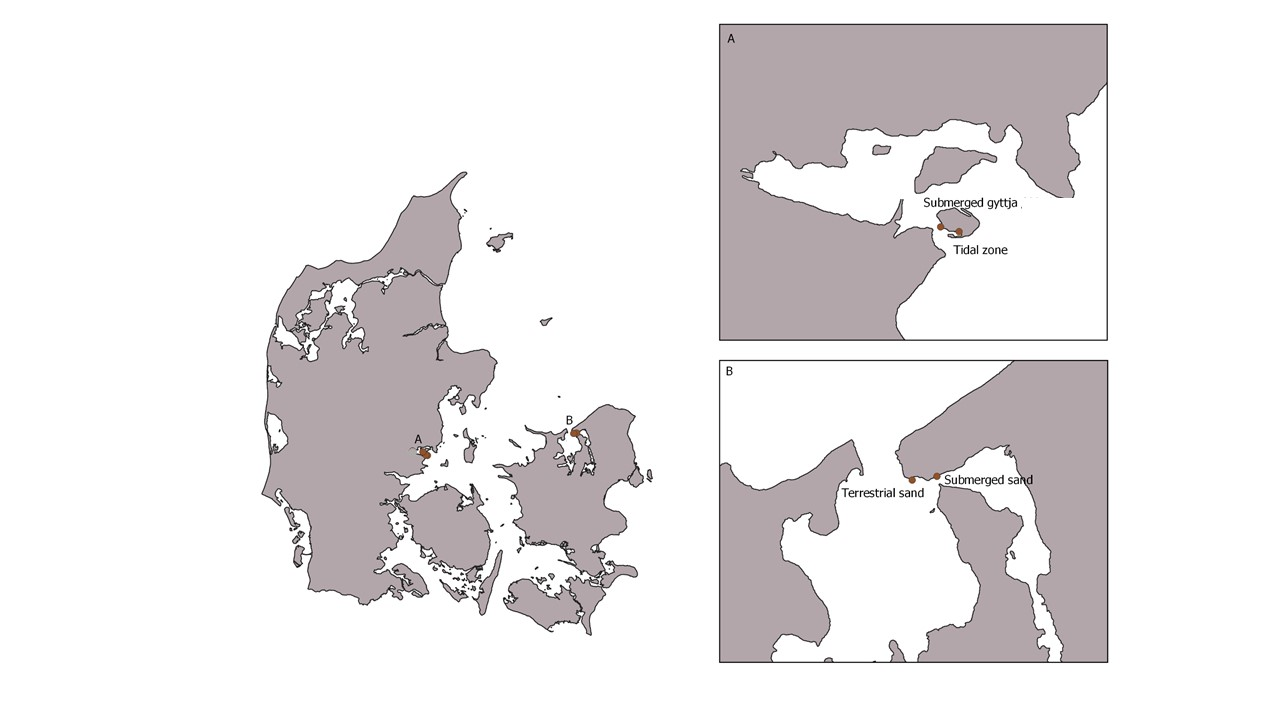

Supplement: S1 Fig — The location of the four different depositional environments are shown on the map of Denmark. The submerged gyttja and tidal zone environments are located on the Island of Hjarnø in the Bay of Horsens in Eatern Jutland, whereas the submerged sand and the terrestrial sand environments are located at the entrance to Isefjord in Northern Zealand. Figure modified from S1 Fig, Eriksen, Matthiesen et al. [25]. (TIF) [file pone.0240512.s001.tif]

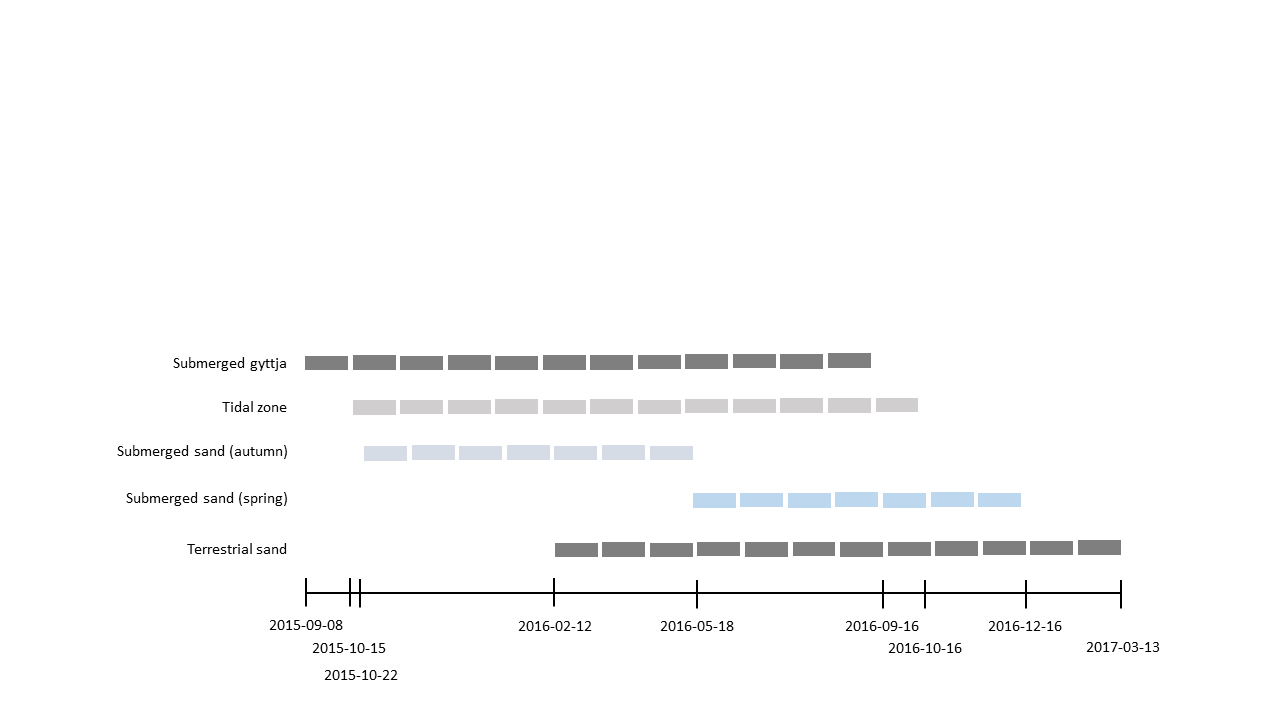

Supplement: S2 Fig — The sampling period for the four environments. Each bar symbolises one month. (TIF) [file pone.0240512.s002.tif]

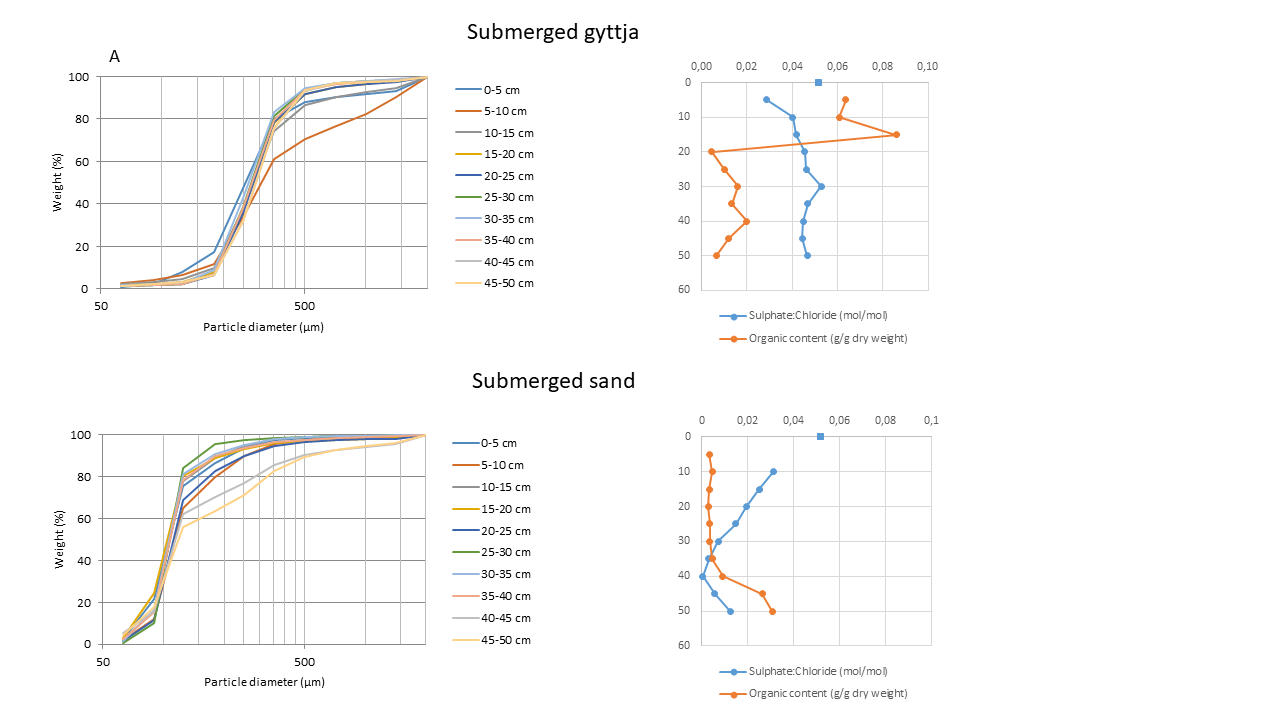

Supplement: S3 Fig — Results from the grain size, organic content and Sulphate:Chloride analysis of the sediments at the two submerged environments. Total organic matter is presented as % of sediment dry weight. The Sulphate:Chloride content is presented as mol/mol. For the submerged gyttja, the visual characterisation showed: 0–18 cm dark brown/grey sandy gyttja, 18–50 cm dark grey sand with some gyttja where the gyttja content decreases with depth. For the submerged sand, the visual characterisation showed: 0–40 cm light grey fine sand with few plant remains, 40–50 cm dark sand with significant amount of plant remains. (TIF) [file pone.0240512.s003.tif]

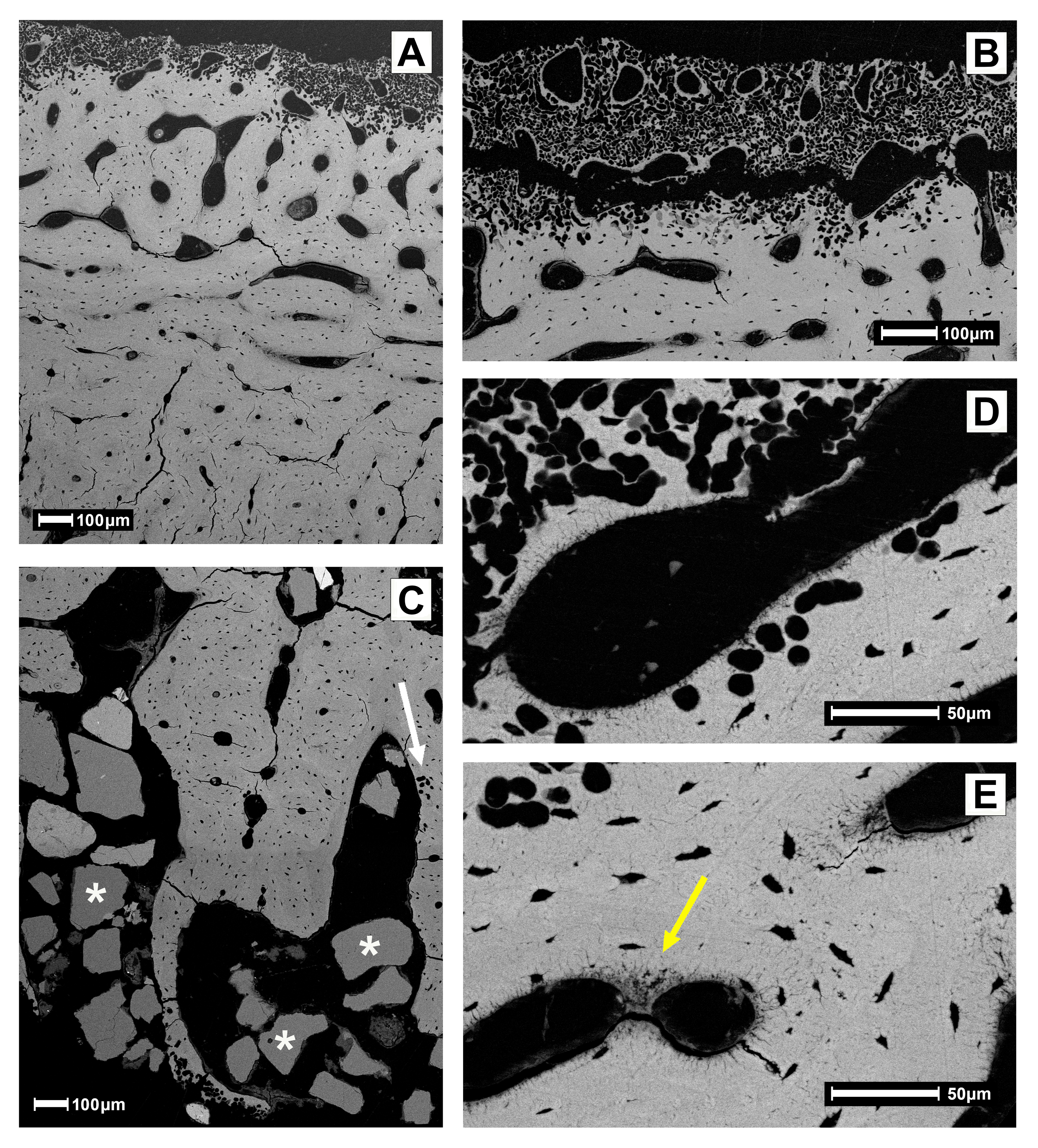

Supplement: S4 Fig — A): Raw bone fragment exposed for one year at the tidal zone (sample ID 8). Extensive Wedl-tunneling is observed on the periosteal surface. B): Tunneling may be more extensive than the images suggest since there have been some obvious losses by exfoliation of tunneled surfaces. All samples deployed for one year at the tidal zone showed a similar type of damage. C): In addition to the severe tunneling into the periosteal surfaces the bones also exhibit some sporadic bioerosion in parts of the spongy bone (white arrow). Sand particles within the spongy bone are indicated by an asterix. D): Detail of Wedl-type tunneling. E): local demineralisation around Haversian canals (yellow arrow). (TIF) [file pone.0240512.s004.tif]

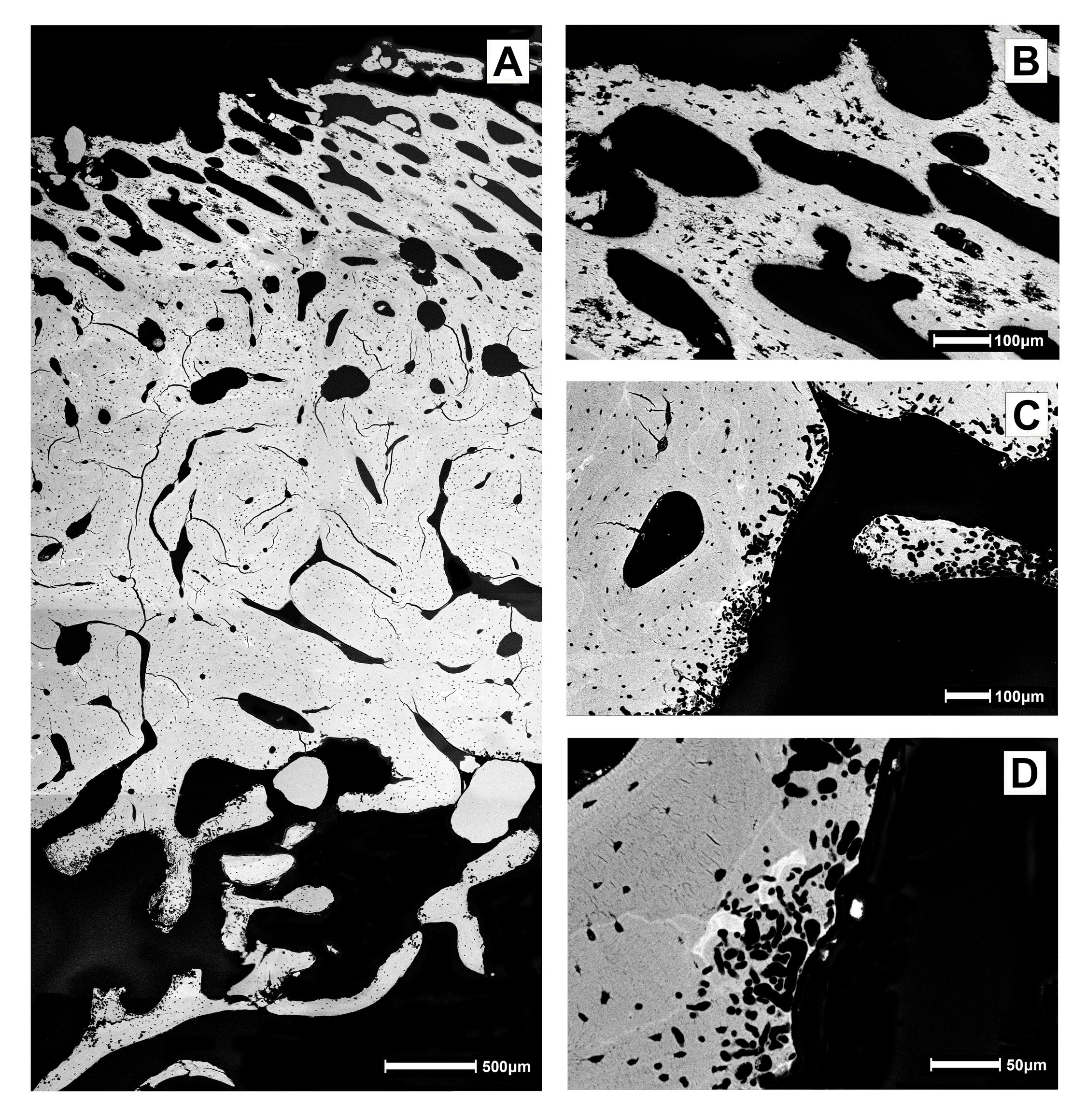

Supplement: S5 Fig — A): Baked bone fragment exposed at the submerged gyttja depositional environment for one year (sample ID 45). B): Chemical demineralisation and enlarged porosity near the periosteal surface has caused surface losses. C): Wedl-tunneling in spongy bone. D): Detail of Wedl-type tunneling. (TIF) [file pone.0240512.s005.tif]

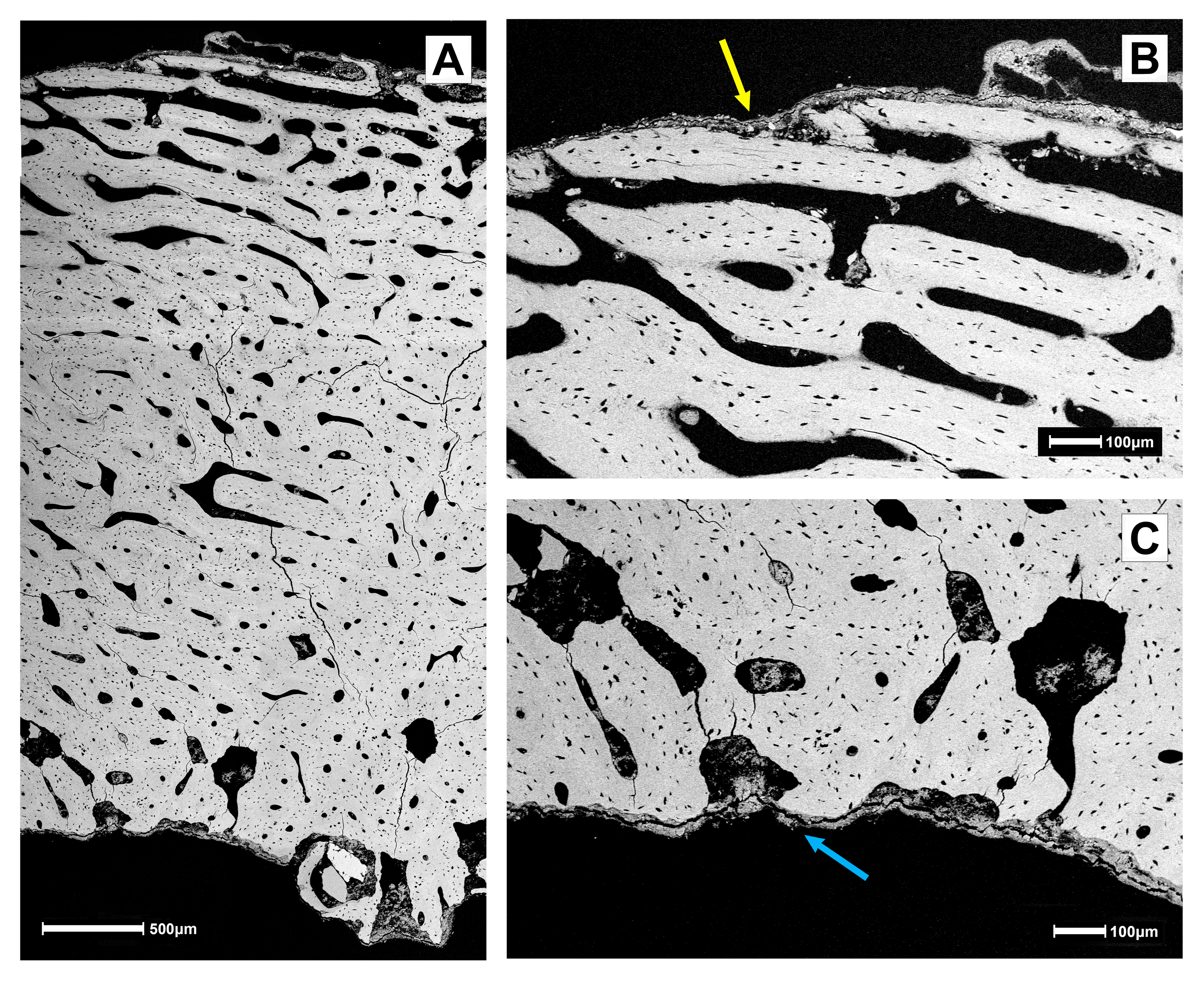

Supplement: S6 Fig — Raw bone fragment deposited at the submerged sand (spring) environment for 28 weeks (Sample ID 16). A): Mosaic showing the whole section. B): Periosteal surface showing adhering fine sediment particles (yellow arrow). C): Endosteal surface showing only minor demineralisation and erosion with adhering sediment (blue arrow). There are no signs of bioerosion in the sample. (TIF) [file pone.0240512.s006.tif]

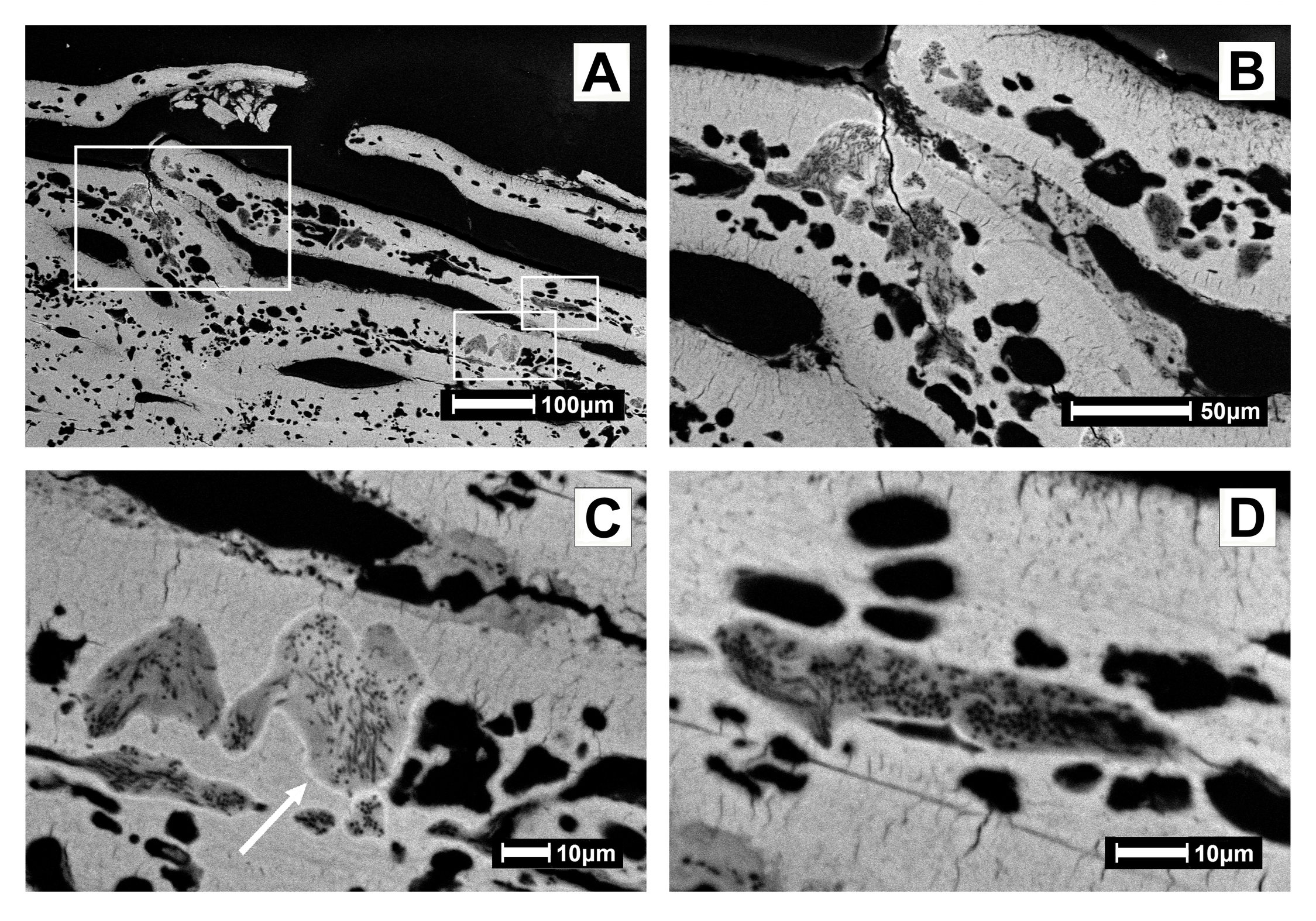

Supplement: S7 Fig — Boiled bone fragment deposited for one year at the terrestrial sand environment (Sample ID 33). A): Non-Wedl, sub-micron microbial tunneling is observed near the periosteal surface and extends to a depth of 200 μm. B): Detail of area indicated by white box in A) showing tunneled regions and large ragged pores where bone has been lost. C): Hypermineralised border around the tunneled region (white arrow). D): The ragged, irregular voids are where both dissolved mineral and bacterially degraded collagen have been washed out of destructive foci. These are quite distinct from the well-delineated Wedl-tunnels seen in S4 and S5 Figs. (TIF) [file pone.0240512.s007.tif]

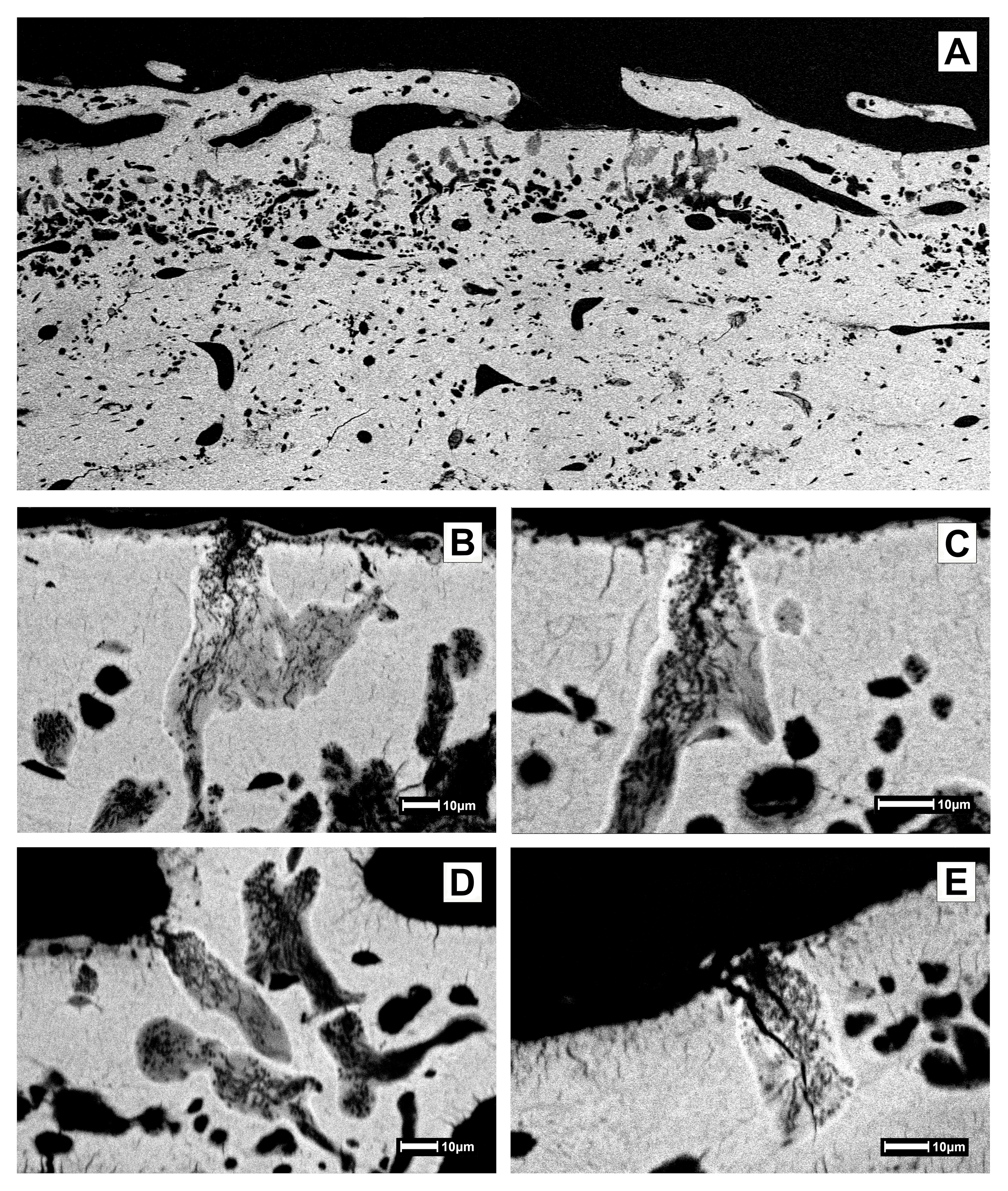

Supplement: S8 Fig — The same boiled bone fragment deposited for one year at the terrestrial sand environment (Sample ID 33) as in S7 Fig. A): Periosteal surface showing numerous foci of non-Wedl, sub-micron microbial tunneling clustered 100–200 microns below the surface. B): Detail of this area showing the periosteal surface where it seems bacteria from the sediment may be entering the bone structure. C,D,E): Very similar morphologies observed at various places along the periosteal surface of the bone fragment. While it may be argued that these images simply show a tunneled zone breaching the surface, the remarkable similarities in size and morphology suggest otherwise. In addition, even in heavily tunneled bones the destructive foci tend to be limited to the interior of the compact bone. The outer 100 microns of the tissues are often quite well preserved. (TIF) [file pone.0240512.s008.tif]

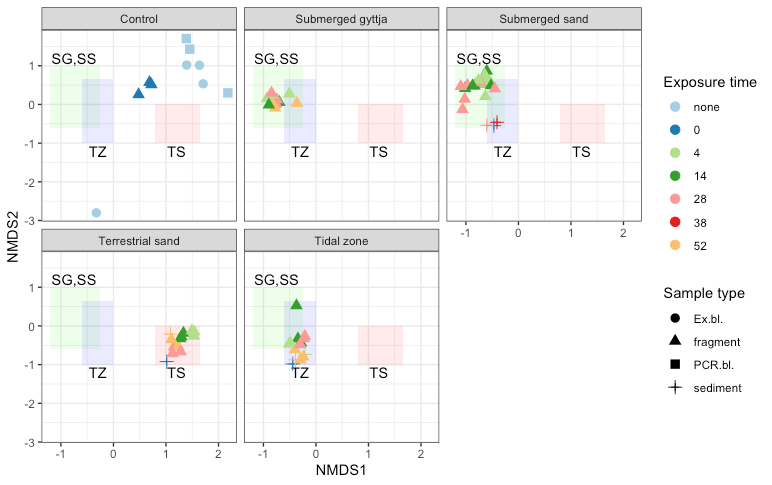

Supplement: S9 Fig — NMDS plot based on beta diversity of the bacterial communities in the bone fragments showing how the extraction and PCR negatives (controls) are clustering away from the rest of the samples. (TIF) [file pone.0240512.s009.tif]

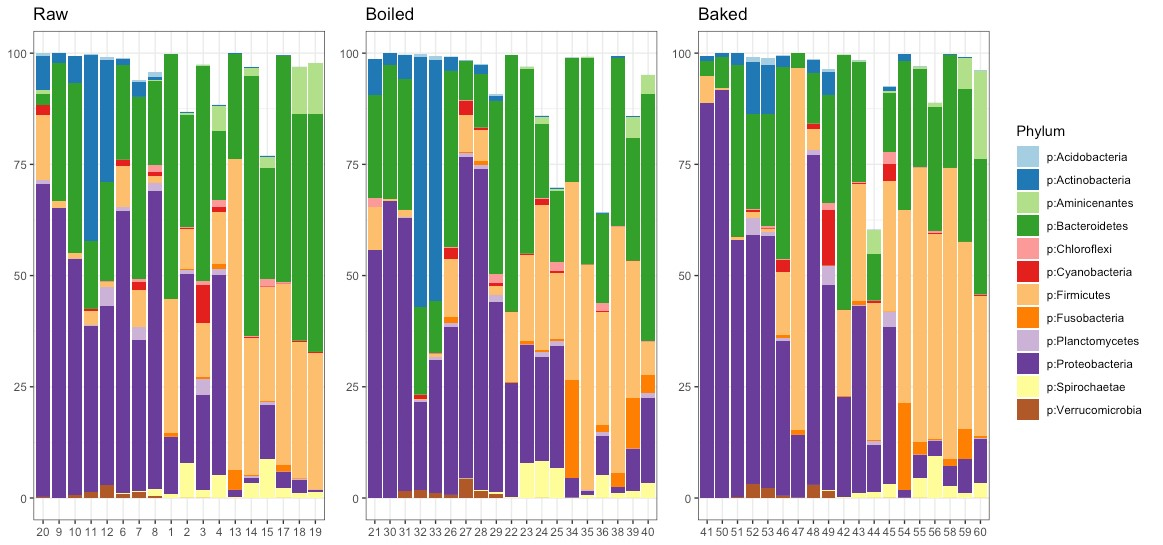

Supplement: S10 Fig — Relative abundance of the 12 most abundant bacterial phyla from the raw, boiled and baked bones respectively. Numbers on the x-axis denote the sample no. from Table 2. (TIF) [file pone.0240512.s010.tif]

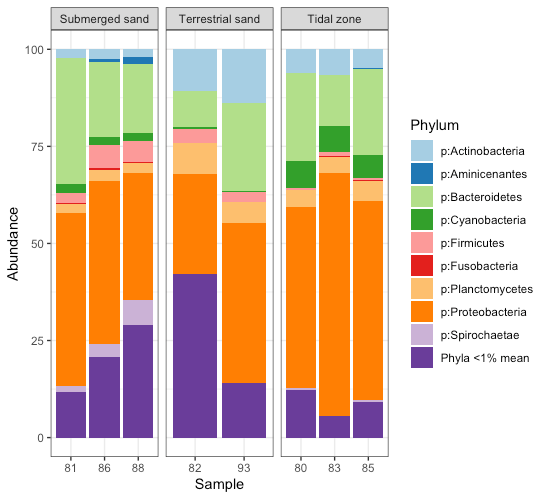

Supplement: S11 Fig — Relative abundance of the most abundant bacterial phyla from the sediment samples. Numbers on the x-axis is the sample no. (Table 2). At the submerged sand samples were collected after 0 (81), 14 (86) and 28 (88) weeks. At the Terrestrial sand samples were collected at 0 (82) and 52 (93) weeks, and at the tidal zone samples were collected at 0 (80), 4 (83) and 52 (85) weeks. (TIF) [file pone.0240512.s011.tif]

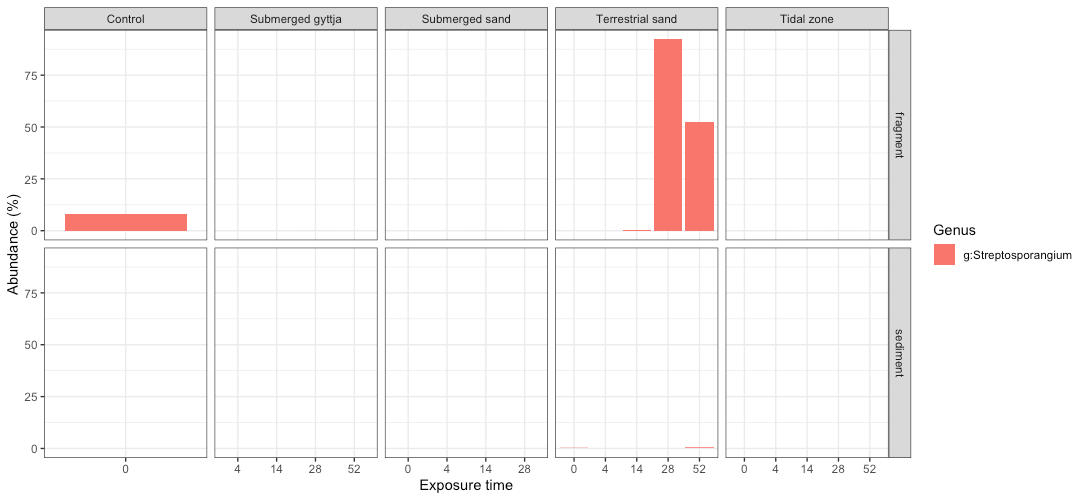

Supplement: S12 Fig — ´control 0’ refers to the unburied bones, where the results from all three unburied bones are shown, however Streptosporangium was absent from the raw and baked unburied bone, thus the results shown here only exhibit the relative amount in the unburied boiled bone fragment. Caution should be taken when assessing the sediment data, as data was not obtained from all environments at all time points (see Table 2). (TIF) [file pone.0240512.s012.tif]

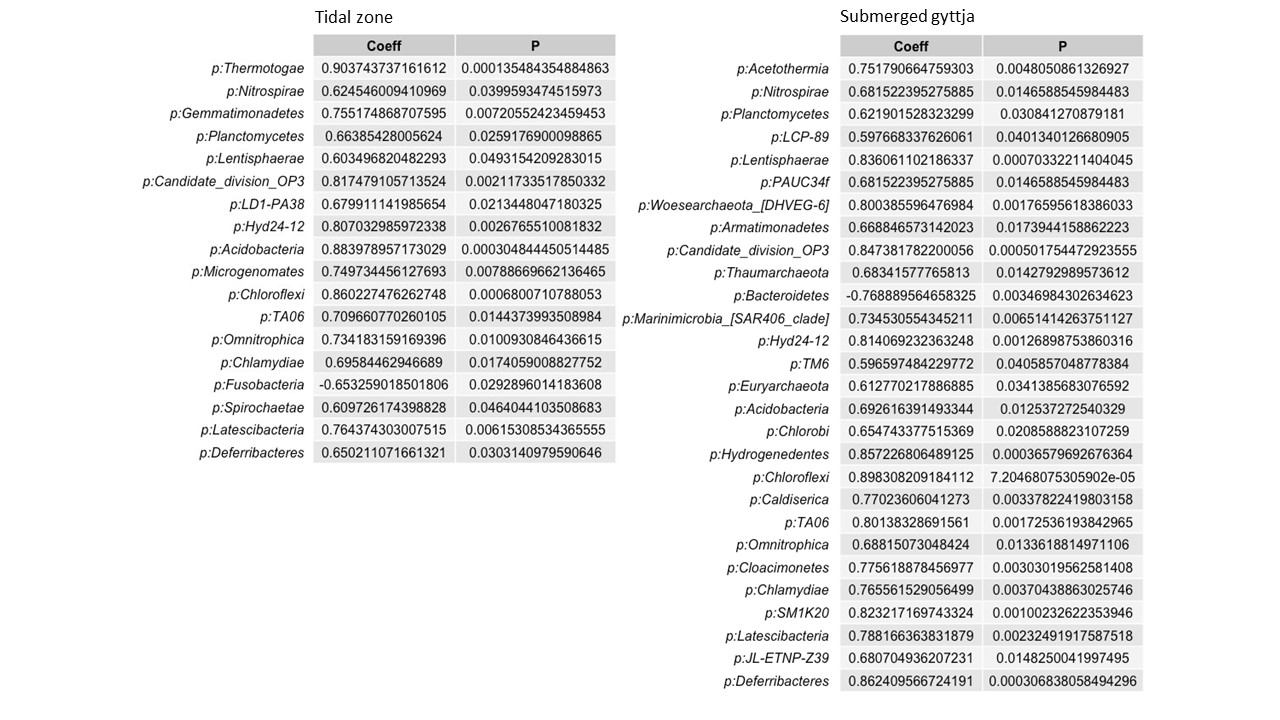

Supplement: S1 Table — Phyla with a significant correlation with exposure time are given for the two marine associated environments in which we observe tunneling on the bones after one year of exposure. (TIF) [file pone.0240512.s013.tif]
